# Network pharmacology evaluation of the active ingredients and potential targets of XiaoLuoWan for application to uterine fibroids

**DOI:** 10.1042/BSR20202342

**Published:** 2020-12-04

**Authors:** Yonghui Yu, Fang Yang, Hong Liu

**Affiliations:** Gynecological Department of Traditional Chinese Medicine, China-Japan Friendship Hospital, Beijing 100029, P.R. China

**Keywords:** calcium signaling pathway, network pharmacology, Traditional Chinese medicine (TCM), uterine fibroids (UFs), XiaoLuoWan (XLW)

## Abstract

XiaoLuoWan (XLW) is a classical formula in traditional Chinese medicine (TCM) that has satisfactory therapeutic effects for uterine fibroids (UFs). However, its underlying mechanisms remain unclear. To elucidate the pharmacological actions of XLW in treating UFs, an ingredient–target–disease framework was proposed based on network pharmacology strategies. The active ingredients in XLW and their putative targets were obtained from the TCM systems pharmacology database and analysis platform (TCMSP) and Bioinformatics Analysis Tool for Molecular mechANism of Traditional Chinese Medicine (BATMAN-TCM) platforms. The known therapeutic targets of UFs were acquired from the DigSee and DrugBank databases. Then, the links between putative XLW targets and therapeutic UF targets were identified to establish interaction networks by Cytoscape. Finally, Gene Ontology (GO) enrichment and Kyoto Encyclopedia of Genes and Genomes (KEGG) pathway analyses of overlapping gene targets were performed in the STRING database and visualized in R software. In total, 9 active compounds were obtained from 74 ingredients, with 71 curative targets predicted in XLW. Moreover, 321 known therapeutic targets were closely related to UFs, with 29 targets overlapping with XLW and considered interacting genes. Pathway enrichment revealed that the calcium signaling pathway was significantly enriched and the mitogen-activated protein kinase (MAPK) signaling pathway, cAMP signaling pathway, cancer and vascular smooth muscle contraction pathways, cGMP-PKG signaling pathway, and AGE-RAGE signaling pathway were closely associated with XLW intervention for UFs. In conclusion, the network pharmacology detection identified 9 available chemicals as the active ingredients in XLW that may relieve UFs by regulating 29 target genes involved in the calcium signaling pathway, MAPK pathway and cAMP pathway. Network pharmacology analyses may provide more convincing evidence for the investigation of classical TCM prescriptions, such as XLW.

## Introduction

Uterine fibroids (UFs), which are also known as leiomyomas or myomas, are the most common benign pelvic tumors and affect 50–60% of women of child-bearing age [1] and 70% of women up to 50 years of age [2]. Most fibroids are asymptomatic, whereas 30–40% of patients may suffer from symptoms, such as pelvic pain, infertility [3] and obstetric complications [4], with 30% of such cases leading to morbidity due to excessive uterine bleeding and pelvic pressure [5]. Current mainstream treatments include surgical procedures and medical therapies [6]. However, associated surgeries, such as hysterectomy or myomectomy, may not increase pregnancy rates [7] or reverse pregnancy loss [8] and may actually increase the risk of mortality [9,10]. Nonextirpative procedures for UFs including uterine artery embolization, focused ultrasound, laparoscopic radiofrequency ablation were reported to show similar outcomes to myomectomy [11]. Drug-based therapy, such as gonadotropin-releasing hormone (GnRH) agonists, which is the only agent for short-term management of UFs approved by Food and Drug Administration (FDA) [12], may cause symptoms associated with false menopause and dormant fertility after long-term intervention [13]. Therefore, novel effective alternatives with fewer side effects must be developed.

Traditional Chinese medicine (TCM), a group of complementary and alternative medicine remedies, has attracted increasing attention in the treatment of UFs [14]. A nationwide population-based study of 35786 newly diagnosed UF patients found that 87.1% of patients had visited TCM clinics and that 61.8% of patients used Chinese herbal remedies [15]. A systematic review revealed that TCM seems to contribute to a decrease in the development of fibroids [16] and a reduction in myoma-related surgeries [17,18]. XiaoLuoWan (XLW), which includes *Radix Scrophulariae* (XuanShen), *Concha Ostreae* (ShengMuLi), and *Bulbus Fritillariae ferganensis* (ZheBeiMu), was first recorded in *Medical Enlightenment from Heart* during the Qing dynasty and is one of the classical and standard TCM formulas for relieving leiomyoma in TCM [19].

*Radix Scrophulariae* (XuanShen) is derived from the roots of *Scrophularia ningpoensis Hemsl* and demonstrates excellent pharmacological activities, such as regulation of immune response and uric acid metabolism as well as protection of heart, liver and neuron functions [20], potentially by affecting mitogen-activated protein kinase (MAPK) [21] and NF-κB pathways [22]. *Bulbus Fritillariae ferganensis* (ZheBeiMu) is a well-known herb that can eliminate phlegm, detoxify carbuncles, and relieve swelling. Modern pharmacological research has found that verticine and peiminine derived from ZheBeiMu can reverse the multidrug resistance of tumor cells, induce cell cycle arrest and apoptosis in malignant cells [23], and regulate inflammatory cytokine the secretion in macrophages via the MAPK pathway [24]. *Concha Ostreae* (ShengMuLi), the crushed shell of Ostrea gigas Thunberg, Ostrea talienwhanensis Crosse, or Ostrea rivularis Gould, could resolve hard lumps, soothe the nerves, and reinforce body fluids according to TCM terminology, and this multimineral and multivitamin marine TCM was found to exert anti-tumor effects, including anti-UF effects via specific medication rules [25].

XLW is now frequently used and has satisfactory therapeutic effects in patients suffering from UFs. Our unpublished study showed that XLW treatment could effectively relieve the symptoms of menorrhagia and pelvic pain caused by UF and produced less obvious side effects after short-term application for 3 months in UF patients. Moreover, a drug serum of XLW may suppress cell proliferation and promote cell apoptosis in cultured human uterine leiomyoma cells *in vitro* according to our unpublished experiment. However, the underlying mechanisms remain unclear. Thus, the pharmacological mechanisms of XLW must be precisely characterized before it can be widely administered to UF patients.

Network pharmacology has recently been developed as a novel strategy to elucidate complex pharmacological problems in new drug discovery [26] because it can provide a holistic view of the mechanisms induced by multiple ingredients [27]. Most TCM prescriptions have numerous ingredients and multiple targets, and the active compounds and potential pathways of some of the TCM remedies that have roles in gynecological diseases have been demonstrated by network pharmacology, including Guizhi Fuling Wan on UFs [28], Erxian Decoction on polycystic ovary syndrome [29], and Cangfu Daotan Decoction for the treatment of polycystic ovary syndrome [30], thus highlighting the pharmacological potential of TCM for women. Here, we used the network pharmacology approach to predict the active ingredients and potential targets of XLW for application to UFs and obtain insights into the mechanisms of XLW in treating UFs.

## Materials and methods

### Database and platform

We identified the chemical ingredients in XLW, gene targets of active ingredients in XLW, and known therapeutic targets of UFs by using online databases and platforms as follows (Table 1).

**Table 1:** Databases and platforms used for the network pharmacology analysis

Database	Full name of database	Sources
TCMSP	TCM systems pharmacology database and analysis platform	https://tcmspw.com/tcmsp.php
BATMAN	Bioinformatics Analysis Tool for Molecular mechANism of Traditional Chinese Medicine	http://bionet.ncpsb.org/batman-tcm
ChemSpider	ChemSpider database	http://www.chemspider.com
UnitProt	UnitProt knowledgebase	https://www.uniprot.org/uniprot
DrugBank	DrugBank database	https://www.drugbank.ca
DigSee	Disease Gene Search Engine with Evidence Sentences	http://gcancer.org/digsee
STRING	STRING: functional protein association networks	https://string-db.org/
Cytoscape	Cytoscape (Version 3.7.1)	https://www.cytoscape.org
Bioinformatics	Bioinformatics database	http://www.bioinformatics.com.cn
R software	R Project for Statistical Computing (Version 3.6.3)	https://www.r-project.org

### Chemical ingredients of each material in XLW

The chemical ingredients in XLW were screened via the TCM systems pharmacology database and analysis platform (TCMSP), Bioinformatics Analysis Tool for Molecular mechANism of Traditional Chinese Medicine (BATMAN-TCM) platform and ChemSpider database and updated on 1 February 2020. The TCMSP database is designed for all 499 Chinese herbs registered in the Chinese pharmacopeia, and each herb contains 12 pharmacokinetic characteristics. This database captures the relationships between herbs, ingredients, diseases, and targets based on identified drug–target networks and drug–disease networks [31]. The BATMAN-TCM platform mainly contributes to understanding the therapeutic mechanisms of TCM by providing valuable clues about gene targets and performing functional analyses of TCM ingredients [32]. The ChemSpider database contains over 26 million entries from hundreds of data sources, and it is valuable in the identification of compounds in natural product samples [33].

### Pharmacokinetic features of ingredients in XLW

The pharmacokinetic properties of active ingredients in XLW were demonstrated in the TCMSP and filtered on the basis of oral bioavailability (OB), Caco-2 permeability (Caco-2), and drug-likeness (DL), which are the three most key indicators of pharmacology. Specifically, the ingredients with OB ≥ 30%, Caco-2 ≥ 0.4, and DL ≥ 0.18 were chosen as candidate ingredients in XLW for further analysis. *Concha Ostreae* (ShengMuLi) is not a herbal medicine that can be found in TCMSP; therefore, its pharmacokinetic information was obtained from the BATMAN-TCM database, with a score cutoff ≥ 20 and an adjusted *P*-value <0.05. The candidate ingredients in XLW were further identified in the ChemSpider database for detailed pharmaceutical information.

### Target exploration of ingredients in XLW

The TCMSP database and BATMAN-TCM platform were used for target exploration of active chemical ingredients in XLW. Components that met the pharmacokinetic criteria above were analyzed online to predict their potential targets, and only predicted targets that had interactions with active ingredients in XLW were selected. The protein names of these selected targets were converted into official gene names via UniProtKB, a popular protein sequence database that consists of information curated by biologists, provides crosslinks to approximately 100 external associated databases, and demonstrates comprehensive protein annotations [34]. Here, we searched UniProtKB with the organism limited to *Homo sapiens* (humans).

### Known therapeutic targets in treating UFs

The known therapeutic targets of UFs were acquired from the DigSee and DrugBank databases. The DigSee database was developed to describe genes that are involved in the development of biological events based on searches of MEDLINE abstracts for evidence-containing sentences [35]. The DrugBank database is one of the most widely used drug resources worldwide and contains detailed drug, drug–target, drug action, and drug interaction information for almost all the FDA-approved drugs [36,37]. As a protocol method, we searched the DigSee database with the keywords ‘uterine fibroids’ and ‘uterine leiomyoma’ to obtain reported gene targets of UFs. In addition, based on the ‘drug–target’ interactions in DrugBank, only drugs that were approved to treat UFs were selected, and their targets were considered therapeutic targets of UFs. After integrating the targets from both the DigSee and DrugBank databases, all the referred therapeutic targets for the treatment of UFs were reserved for further network analysis.

### Protein–protein interaction

To clarify the connection of target proteins involved with both XLW and UFs, we intersected the potential targets of XLW and UF-related therapeutic targets, and the overlapping targets were considered XLW-treated targets of UFs. A protein–protein interaction (PPI) network analysis of these overlapping targets was performed using the STRING database, a functional protein association networks platform [38]. The organism type was selected as *Homo sapiens* (humans), the minimum required interaction score was set with a medium confidence = 0.4, and the default setting was retained for other parameters. The ‘string_interactions.tsv’ file was downloaded for network visualization and the ‘enrichment.Component.tsv’, ‘enrichment.Function.tsv,’ ‘enrichment.Process.tsv,’ and ‘enrichment.KEGG.tsv’ files were exported as the results of Gene Ontology (GO) enrichment and Kyoto Encyclopedia of Genes and Genomes (KEGG) pathway analyses.

### Network visualization and enrichment analysis

Network visualization was performed using Cytoscape software (version 3.7.1) [39], and the interactions of potential XLW targets and therapeutic targets of UFs were constructed using the links between them. Data from the STRING platform were imported into Cytoscape to visualize the PPI network. In the network, nodes represent the target proteins and edges represent the interaction between proteins. There were four important properties in the Cytoscape network that were quantified to screen the putative targets for topological importance: ‘degree,’ ‘betweenness,’ ‘closeness,’ and ‘coreness.’ GO enrichment and KEGG pathway analyses are important methods used to annotate the functional characteristics of target genes. We performed the enrichment analysis in the STRING platform and visualized the results by R software (version 3.6.3) [40,41].

## Results

### Active ingredients in XLW

**Figure 1:**
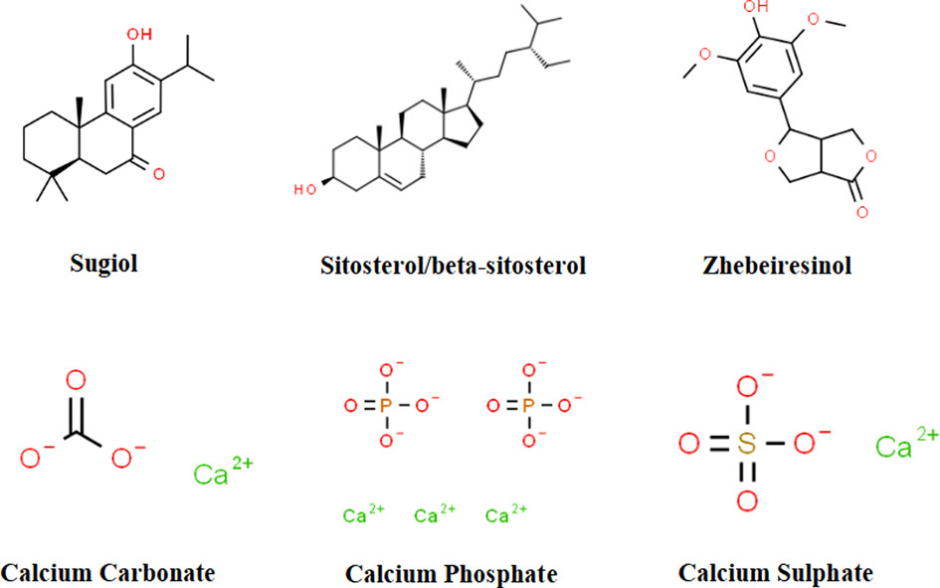
Chemical structural formulas of active ingredients in XLW Aluminum and silicon are not shown in this figure because they are chemical elements.

A total of 74 chemical ingredients in XLW were retrieved from the TCMSP and BATMAN-TCM databases: 47 in *Radix Scrophulariae* (XuanShen), 17 in *Bulbus Fritillariae ferganensis* (ZheBeiMu), and 10 in *Concha Ostreae* (ShengMuLi). After eliminating the repeated and invalid ingredients with unqualified pharmacokinetic conditions, 9 of these chemicals were identified as candidate components (Table 2): 3 in *Radix Scrophulariae* (XuanShen), 2 in *Bulbus Fritillariae ferganensis* (ZheBeiMu), and 5 in *Concha Ostreae* (ShengMuLi). The chemical information for the identified ingredients was recorded from the ChemSpider database (Table 2 and Figure 1).

**Table 2:** Chemical information for the active ingredients in XLW

TCM materials		Active ingredients	Chemical information		
Latin names	Chinese names		ID	Mass (Da)	Formula
*Radix Scrophulariae*	XuanShen	Sugiol	84979	300.435	C_20_H_28_O_2_
		β-Sitosterol	192962	414.386	C_29_H_50_O
		Sitosterol	192962	414.386	C_29_H_50_O
*Bulbus Fritillariae Ferganensis*	ZheBeiMu	β-Sitosterol	192962	414.386	C_29_H_50_O
		Zhebeiresinol	4474746	280.273	C_14_H_16_O_6_
*Concha Ostreae*					
	ShengMuLi	Aluminum	4514248	26.982	Al
		Calcium carbonate	9708	100.087	CCaO_3_
		Calcium phosphate	22864	310.177	Ca_3_O_8_P_2_
		Calcium sulphate	22905	136.141	CaO_4_S
		Silicon	4574465	28.086	Si

### Target exploration of active ingredients in XLW

**Figure 2:**
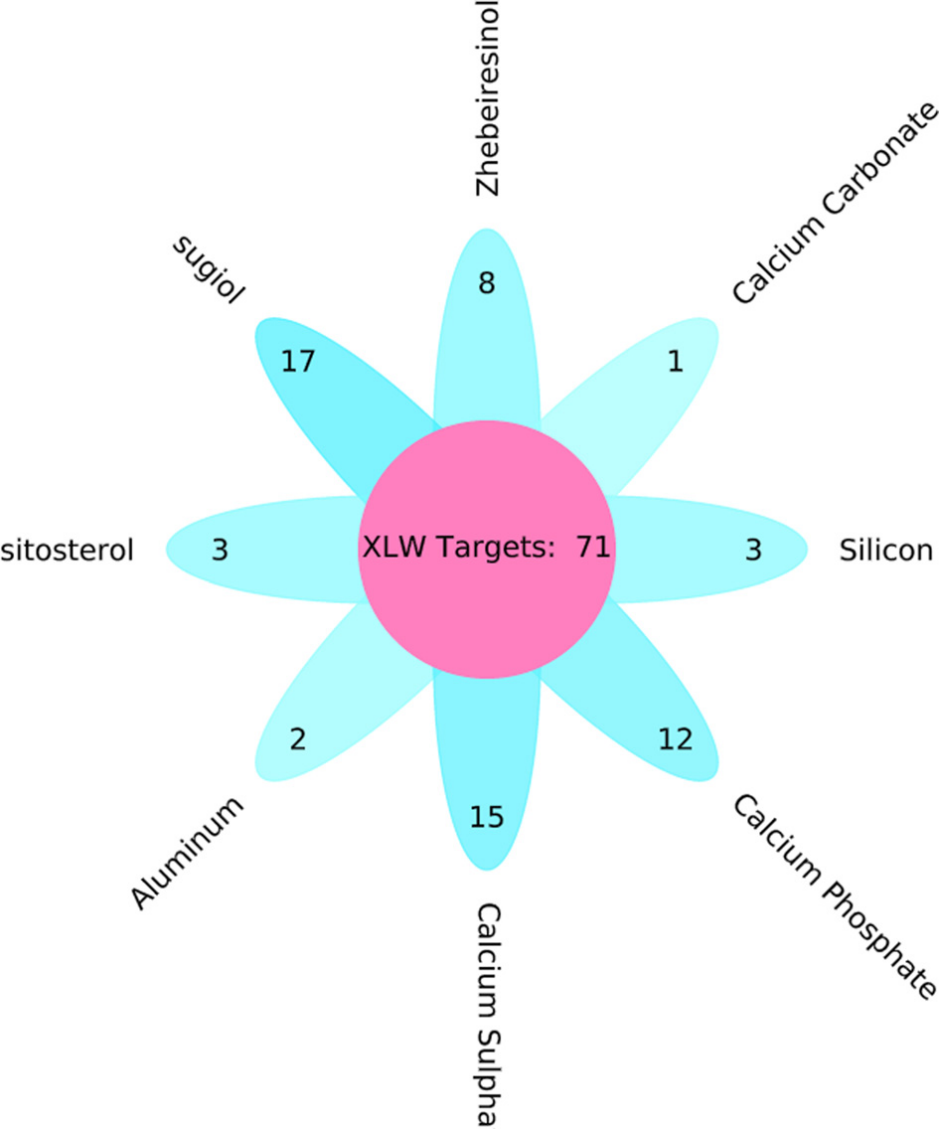
Number of potential targets for each identified active ingredient in XLW shown by a flower plot The petals show the number of target genes found in each identified ingredients of XLW, and the center shows the total gene number of XLW. By the way, sitosterol and β-sitosterol have the same gene targets.

A total of 131 potential targets from the active ingredients in XLW were retrieved from the TCMSP and BATMAN-TCM databases: 58 targets of *Radix Scrophulariae* (XuanShen), 46 targets of *Bulbus Fritillariae ferganensis* (ZheBeiMu), and 27 targets of *Concha Ostreae* (ShengMuLi). After elimination of overlapping targets, 71 targets were obtained (Figure 2) and the protein and gene names were identified in UniProtKB, in which the organism was limited to *H. sapiens* (humans). Detailed information on the active ingredients and putative targets of XLW is provided in Supplementary Tables S1 and S2.

### UF-related target network

**Figure 3:**
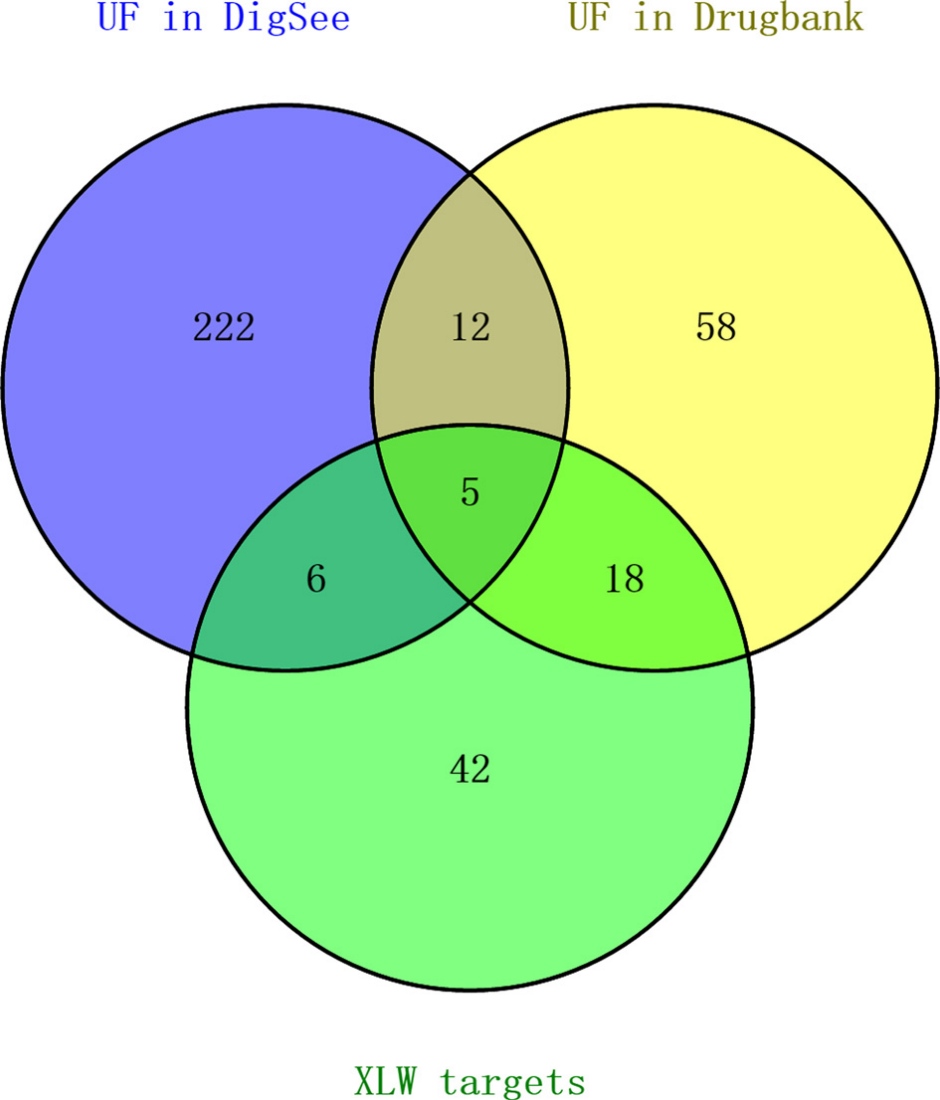
Number of XLW-targeted UF genes shown by a Venn diagram The blue and yellow circles represent UF targets found in the DigSee database and DrugBank database, respectively. The green circle represents the identified XLW targets.

The DigSee and DrugBank databases were searched using the keywords ‘uterine fibroids’ and ‘uterine leiomyoma’ as the keywords to explore the target genes of UFs. In the DigSee database, 245 known therapeutic genes were finally included after eliminating the duplicates. In the DrugBank database, 69 approved drugs with 146 targets were included and 93 targets were chosen for treating UFs after eliminating duplicates. Finally, after integrating the two databases, 321 known therapeutic targets for UFs were used for further data analysis. Detailed information on all the identified known therapeutic targets of UFs is provided in Supplementary Table S3. After comparing the 321 UF-associated targets with the 71 XLW-related targets, 29 common targets (approximately 9.03% of disease targets) were considered effective targets for XLW intervention in UFs (Figure 3).

### PPI network analysis

**Figure 4:**
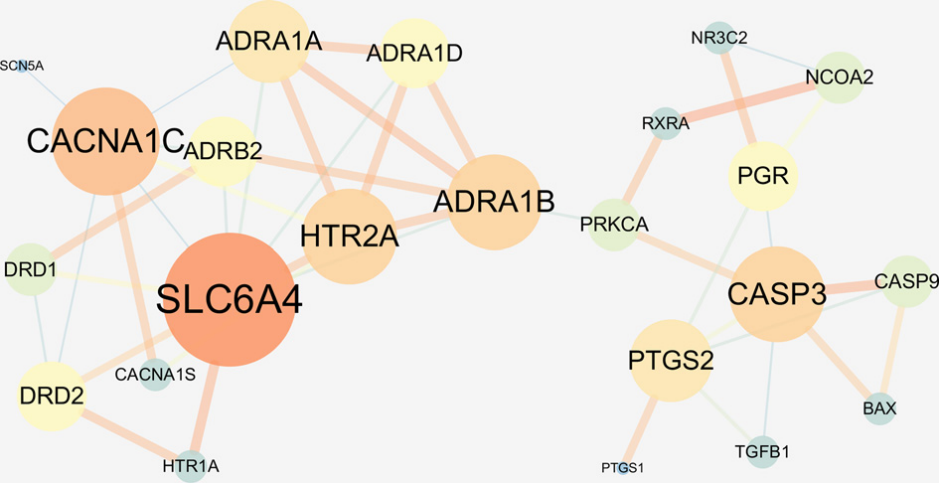
PPI network of targets for XLW in treating UFs The freedom increases from yellow to blue, larger nodes suggest a higher degree, and thicker edges suggest stronger interactions.

The 29 putative XLW-treated UF targets were imported into the STRING database to obtain the interaction analysis of these proteins and then imported into Cytoscape (version 3.7.2) to perform and visualize the PPI network (Figure 4). There were 23 hub nodes and 86 connected edges, the average node degree was 3.74, the average shortest path length was 3.13, and the average clustering coefficient was 0.53. The degrees of freedom increase from yellow to blue, larger nodes suggest a higher degree, and thicker edges suggest stronger interactions. Detailed information on the topological features of these hubs is provided in Supplementary Table S4.

### Network construction of XLW action in treating UFs

**Figure 5:**
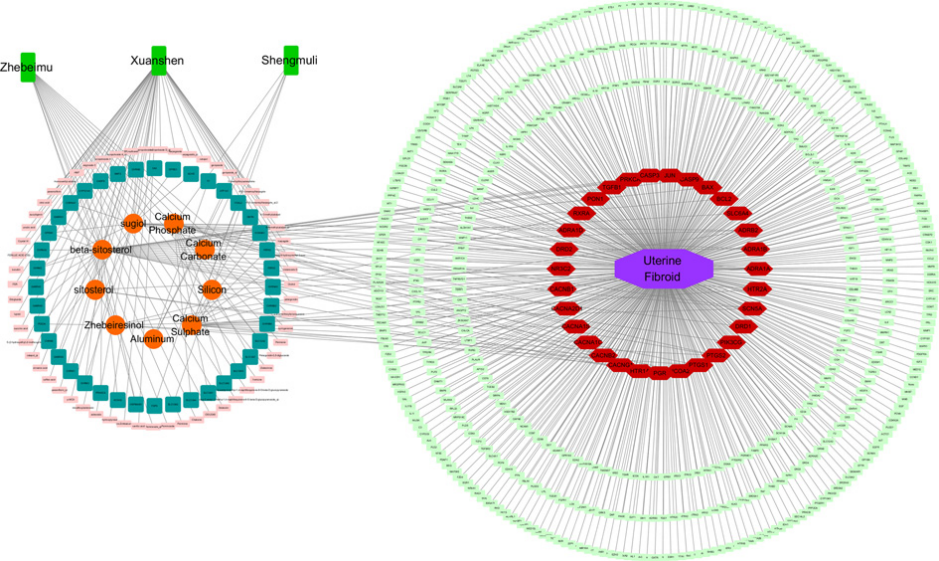
Network connection of ‘XLW–ingredients–targets–UFs’ in terms of the therapeutic mechanisms involving XLW treatment of UFs The grass-green rectangle, orange ellipse, pink rectangle, and dark-green rectangle nodes represent TCM materials in XLW, active ingredients in XLW, inactive ingredients in XLW, and XLW-related targets, respectively (left). The purple octagon, light green rectangle, and red hexagon nodes represent disease, UF-related targets, and the common targets of XLW–UFs, respectively (right).

Finally, Cytoscape (version 3.7.1) software was used to combine the XLW–UF target network and the PPI network and visualize the ‘XLW–ingredient–target–UF’ interaction network (Figure 5). The results showed that the 3 TCM materials in XLW with 9 active ingredients had 29 putative targets in treating UFs, with 23 targets presenting interactions with other targets, including SLC6A4, CACNA1C, CASP3, ADRA1B, HTR2A, ADRA1A, PTGS2, ADRA1D, DRD2, PGR, ADRB2, NCOA2, CASP9, DRD1, PRKCA, RXRA, HTR1A, NR3C2, CACNA1S, BAX, TGFB1, PTGS1, and SCN5A.

### GO enrichment and KEGG pathway analyses

**Figure 6:**
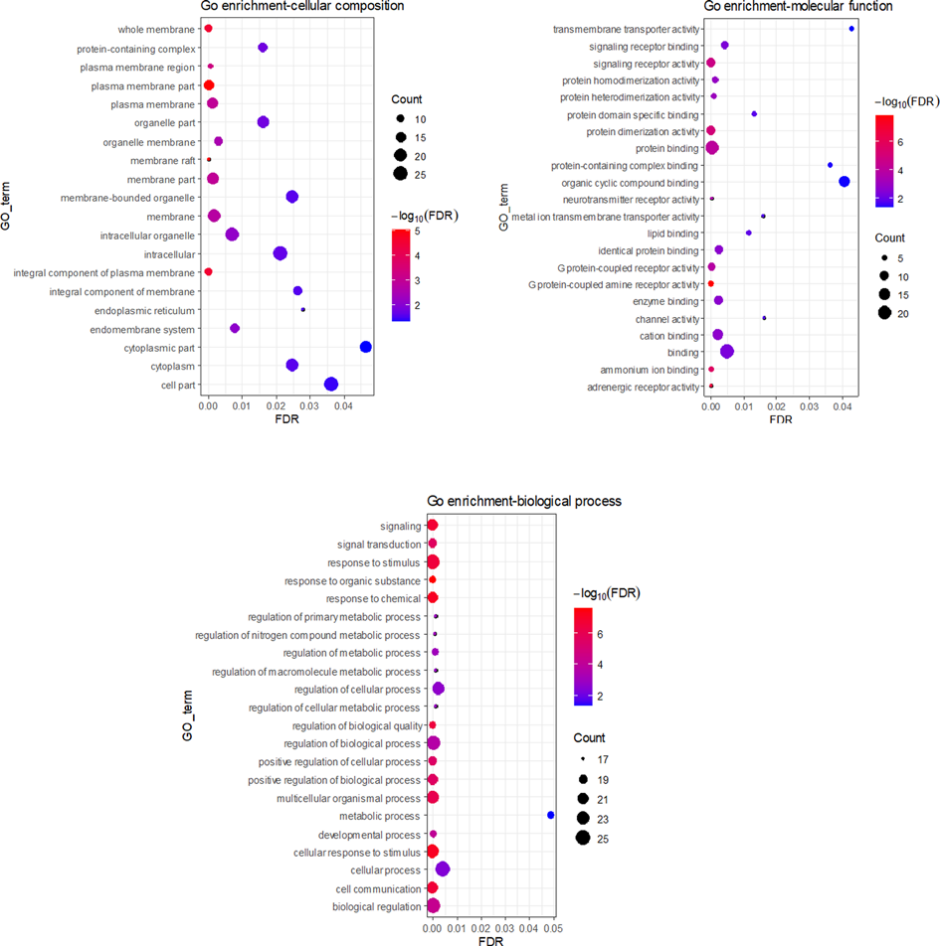
GO enrichment analysis of therapeutic targets of XLW in treating UFs The negative FDR value of the enrichment analysis increases from blue to red, the gene count enriched in GO term increases from small to large, that is larger nodes suggest more enriched genes. Abbreviation: FDR, false discovery rate.

To further elucidate the biological effects of the UF treatment with XLW, we performed GO enrichment and KEGG pathway analyses of the 29 UF-related potential therapeutic target genes in the STRING database. Detailed information on the results of the enrichment analysis is provided in Supplementary Table S5. The GO annotation and enrichment were conducted from three aspects: cellular composition (C), molecular function (F), and biological process (P). The most enriched terms were compiled as bubble diagrams by R software (Figure 6).

Specifically, the top 10 enriched terms in GO-cellular composition were cell parts (Count = 25, false discovery rate (FDR) = 0.0363), intracellular parts (Count = 24, FDR = 0.0212), intracellular organelle (Count = 23, FDR = 0.0069), cytoplasm (Count = 21, FDR = 0.0247), membrane-bounded organelle (Count = 21, FDR = 0.0247), membrane (Count = 20, FDR = 0.0017), organelle parts (Count = 19, FDR = 0.0159), membrane parts (Count = 18, FDR = 0.0011), cytoplasmic parts (Count = 18, FDR = 0.0464), and plasma membrane (Count = 16, FDR = 0.0011).

The top 10 enriched terms in GO-molecular function were binding (Count = 23, FDR = 0.0048), protein binding (Count = 20, FDR < 0.0001), cation binding (Count = 14, FDR = 0.0020), organic cyclic compound binding (Count = 13, FDR = 0.0407), protein dimerization activity (Count = 11, FDR < 0.0001), signaling receptor activity (Count = 11, FDR < 0.0001), enzyme binding (Count = 10, FDR = 0.0022), identical protein binding (Count = 9, FDR = 0.0022), G protein-coupled receptor activity (Count = 8, FDR = 0.0002), and signaling receptor binding (Count = 8, FDR = 0.0041).

The top 10 enriched terms in GO-biological process were biological regulation (Count = 25, FDR < 0.0001), cellular process (Count = 25, FDR = 0.0042), response to stimulus (Count = 24, FDR < 0.0001), regulation of biological process (Count = 24, FDR = 0.0002), cellular response to stimulus (Count = 23, FDR < 0.0001), multicellular organismal process (Count = 22, FDR < 0.0001), regulation of cellular process (Count = 22, FDR = 0.0021), signaling (Count = 21, FDR < 0.0001), cell communication (Count = 21, FDR < 0.0001), response to chemical (Count = 20, FDR < 0.0001), and positive regulation of biological process (Count = 20, FDR < 0.0001).

**Figure 7:**
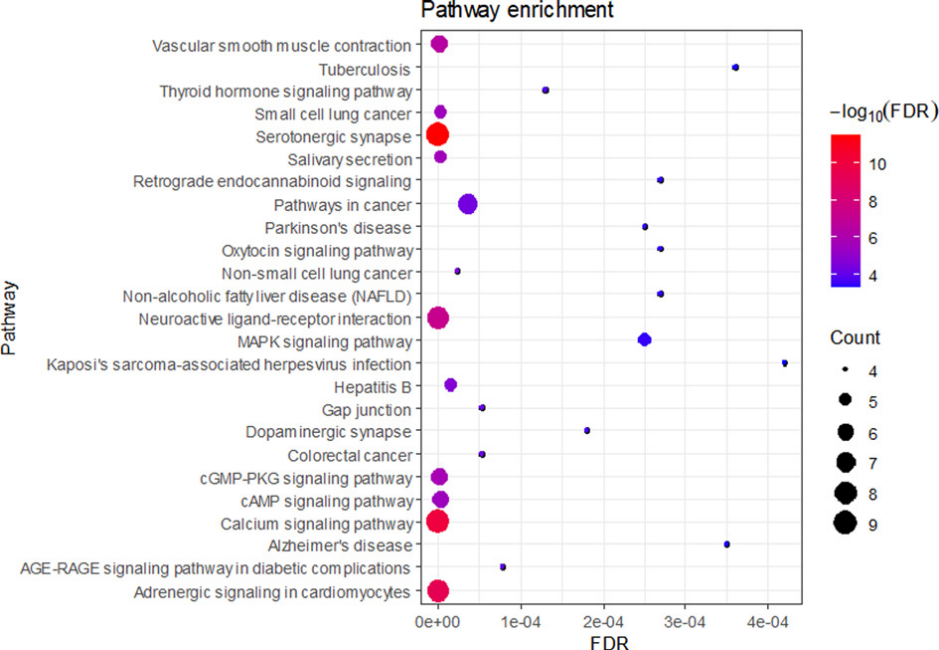
KEGG pathway analysis of the therapeutic targets of XLW in treating UFs The negative FDR value of the enrichment analyse increases from blue to red, the gene count enriched in KEGG pathway increases from small to large, that is larger nodes suggest more enriched genes.

**Figure 8:**
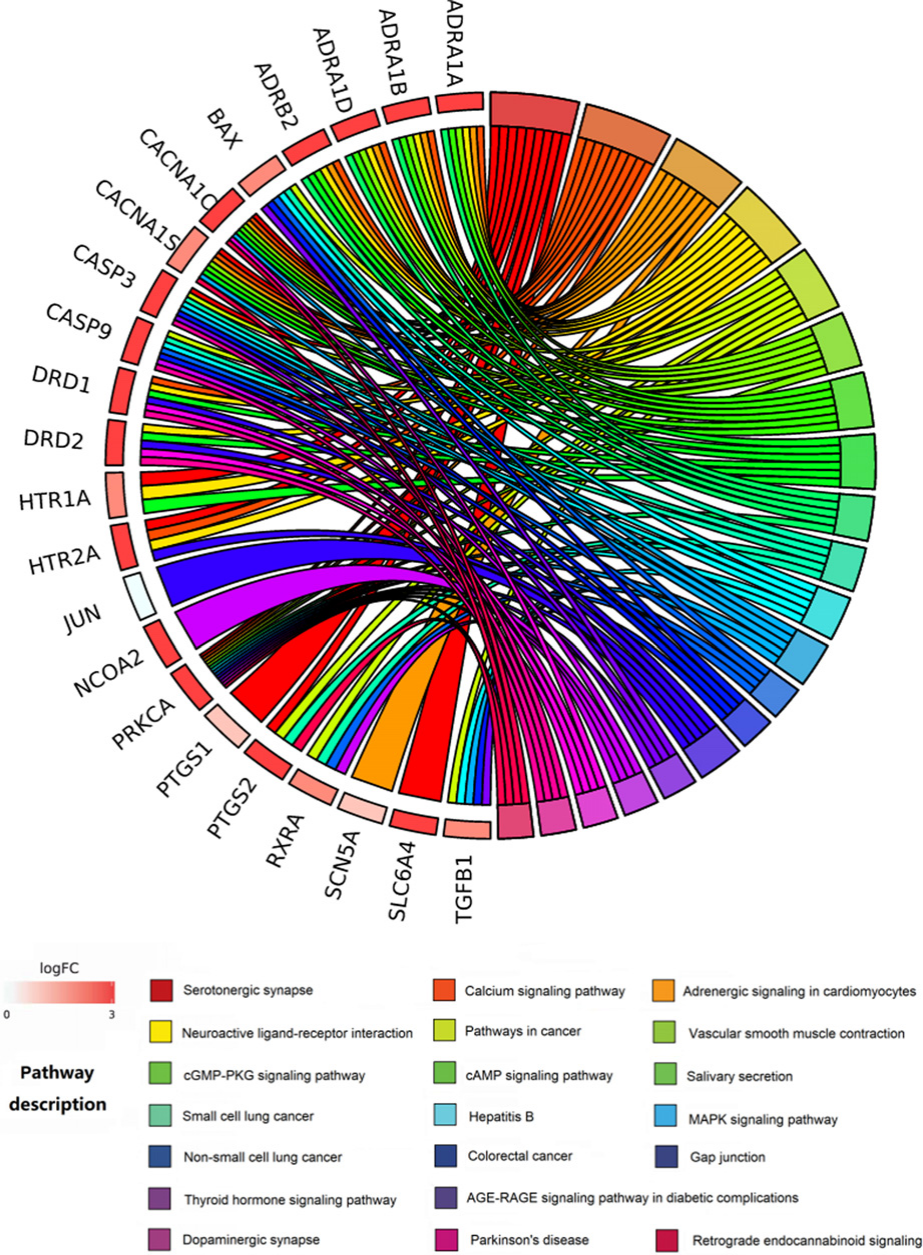
Chordal graph of the KEGG pathway analysis of the therapeutic targets of XLW in treating UFs The different shades of red rectangle nodes around the chordal graph represent the therapeutic targets, the varicolored square nodes around the chordal graph represent the KEGG pathways, and the chordal lines represent the ownership of targets and pathways.

In total, 85 signaling pathways were significantly enriched through the KEGG pathway enrichment analysis. In the bubble diagram of the top 25 pathways (Figure 7), the size and color of the nodes were determined based on the counts and FDRs of the related pathways. The chordal graph was constructed according to the connection between target genes and enriched pathways (Figure 8). The top 15 terms in the KEGG pathway were enriched in serotonergic synapse (Count = 9, FDR < 0.0001), calcium signaling pathway (Count = 9, FDR < 0.0001), adrenergic signaling in cardiomyocytes (Count = 8, FDR < 0.0001), neuroactive ligand–receptor interaction (Count = 8, FDR < 0.0001), pathways in cancer (Count = 7, FDR < 0.0001), vascular smooth muscle contraction (Count = 6, FDR < 0.0001), cGMP-PKG signaling pathway (Count = 6, FDR < 0.0001), cAMP signaling pathway (Count = 6, FDR < 0.0001), salivary secretion (Count = 5, FDR < 0.0001), small cell lung cancer (Count = 5, FDR < 0.0001), hepatitis B (Count = 5, FDR < 0.0001), MAPK signaling pathway (Count = 5, FDR < 0.0001), non-small cell lung cancer (Count = 4, FDR < 0.0001), colorectal cancer (Count = 4, FDR < 0.0001), and gap junction (Count = 4, FDR < 0.0001).

## Discussion

TCM products have been widely used for UFs in China for thousands of years. Moreover, the pharmacological actions of many TCM herbs or pairs in myomagenesis have been confirmed recently. The *Cinnamon Twig* and *Poria Pill* (GuiZhi FuLing Wan) formula was the most frequently prescribed for UFs in TCM clinics, and it has beneficial effects on both fibroid characteristics and sex hormones *in vivo* [42] and can efficiently inhibit the survival of leiomyoma cells *in vitro* [43]. XLW, including *Radix Scrophulariae* (XuanShen), *Concha Ostreae* (ShengMuLi), and *Bulbus Fritillariae ferganensis* (ZheBeiMu), a classical and standard formula for relieving scrofula in TCM, is now frequently used in UFs and has satisfactory therapeutic effects in the clinic. However, the efficacy of XLW has not been confirmed in standard clinical trials and the underlying mechanisms are not entirely clear, thus leaving obstacles to its popularization and delaying benefits to more UF patients.

Recently, a network pharmacology approach has provided a new paradigm for driving TCM from experience-based medicine to evidence-based medicine [44], which could provide more convincing clues to TCM pharmacological research. This approach is effective for establishing the interaction network of chemical compounds, proteins or genes, and specific diseases [45]. Moreover, network pharmacology reveals the regulation of small molecules in a high-throughput manner and would thus be efficient for the analysis of multidrug combinations, especially for TCM preparations and worldwide ethnomedicine, whose therapeutic effects occur via targeting of multiple molecules in the human body [46]. In this study, we elucidated the potential mechanisms of XLW in treating UFs via network pharmacology strategies and found that the calcium signaling pathway is significantly enriched and the MAPK signaling pathway, cAMP signaling pathway, pathways in cancer and vascular smooth muscle contraction, cGMP-PKG signaling pathway, and AGE-RAGE signaling pathways are closely associated in XLW intervention for UFs.

The calcium signaling pathway has been highlighted in XLW-treated UFs, and it involves six calcium voltage-gated channel genes: CACNA1C, CACNA1S, CACNA2D1, CACNB1, CACNB2, and CACNG1. The relationship between calcium channels and UF pathogenesis has been reported in previous studies. An assessment of cell membrane calcium channel proteins in UF tissues showed that the expression patterns of the calcium channel proteins TRPC1 and TRPM7 in UF tissues were different from that in adjacent smooth muscle tissues and the *in vitro* modification of TRPC1 and TRPM7 expression significantly affected the proliferation of uterine leiomyoma cells [47]. Inhibition of store-operated Ca^2+^ channels was demonstrated to induce the second sustained [Ca^2+^]i and suppress cell proliferation in uterine leiomyoma cells [48]. Voltage-gated calcium channels are also involved in the proliferation of uterine leiomyoma cells via their participation in apoptotic calcium release [49]. XLW may affect the expression of calcium channel proteins, influence calcium homeostasis in uterine leiomyoma cells, and consequently regulate cell survival.

The MAPK pathways are well known for their important roles in myomagenesis, and they have targets in estrogen-dependent benign gynecological disorders, such as UFs [50]. The binding of estrogen to receptors initiates a cascade of molecular events, including MAPK activation, Ras/Raf activation, and MEK phosphorylation [51], which lead to the transcription of target genes associated with cell proliferation, survival, and apoptosis. Moreover, UFs are characterized by increased levels of extracellular matrix (ECM), including collagens, fibronectin, laminins and proteoglycans, in the interstitial uterine tissue [52]. Pathological factors can induce the activation of the MAPK-regulated integrin-Rho/ERK pathway [53], resulting in cellular responses and ECM deposition that are involved in altered bidirectional signaling between leiomyoma cells and the ECM. XLW may intervene with UFs via regulation of the MAPK cascade, leading to anti-fibroid effects involving cell proliferation, apoptosis, and ECM production.

cAMP is a vital second messenger that regulates various cellular functions, including inflammation, lipid metabolism, and cell differentiation, by affecting the expression and functions of important genes or proteins [54]. The cAMP pathway was proven to be related to psychological stress, which can activate the neuroendocrine system and induce the secretion of catecholamines (CAs) [55], which promote emotion-related illness by the adrenal receptor (AR)-regulated cAMP-PKA signaling pathway. In cultured uterine leiomyoma cells, AR agonists were demonstrated to increase the expression levels of estrogen receptor (ER), progesterone receptor (PR), vascular endothelial growth factor (VEGF), and fibroblast growth factor (FGF) via cAMP-dependent signaling pathways to influence uterine myomagenesis [56]. Therefore, XLW may relieve mental stress-induced UF development by regulating cAMP pathways.

As complex biological processes and complicated signaling are involved in UFs [57], therapeutic strategies that have effects on multiple targets and pathways could be regarded as alternative approaches to managing UFs, such as TCM prescriptions with combined materials. Although XLW only contains three TCM materials, it shown to have anti-fibroid effects through multiple targets and multiple pathways after the network pharmacologic analysis in this study. Further research is urgently needed to verify our hypothesis that the anti-fibroid effects of XLW were related to its regulation of the calcium signaling pathway, MAPK pathway and cAMP pathways. The strong and vigorous potential of XLW in treating UFs may promote pharmacological investigations of simple herbal formulas that are more efficient and have fewer side effects when used to treat intractable human diseases.

## Supplementary Material

Supplementary Tables S1-S5

## Data Availability

The data used to support the findings of the present study are included within the Supplementary Materials.

## Abbreviations

AR: adrenal receptor
BATMAN-TCM: Bioinformatics Analysis Tool for Molecular mechANism of Traditional Chinese Medicine
CA: catecholamine
DL: drug-likeness
ECM: extracellular matrix
ER: estrogen receptor
FDA: Food and Drug Administration
FDR: false discovery rate
FGF: fibroblast growth factor
GnRH: gonadotropin-releasing hormone
GO: Gene Ontology
KEGG: Kyoto Encyclopedia of Genes and Genomes
MAPK: mitogen-activated protein kinase
OB: oral bioavailability
PPI: protein–protein interaction
PR: progesterone receptor
TCM: traditional Chinese medicine
TCMSP: TCM systems pharmacology database and analysis platform
UF: uterine fibroid
VEGF: vascular endothelial growth factor
XLW: XiaoLuoWan
