# Supplementary material for: Network pharmacology evaluation of the active ingredients and potential targets of XiaoLuoWan for application to uterine fibroids
Source: Biosci Rep. 2020 Dec 4;40(12):BSR20202342. doi: 10.1042/BSR20202342 (PMC7724689; doi:10.1042/BSR20202342)
Supplement: Supplementary Tables S1-S5 [file BSR-2020-2342_supp.pdf]

Table S1 Detailed information about active ingredients in XLW

XLW	Molecule ID	Molecule Name	MW	OB (%)	Caco-2	DL
Xuanshen	MOL000114	vanillic acid	168.16	35.47	0.43	0.04
Xuanshen	MOL001301	cis-Zimtsaeure	148.17	38.19	0.91	0.03
Xuanshen	MOL000141	hydroxytyrosol	154.18	57.57	0.3	0.03
Xuanshen	MOL003333	acteoside	624.65	2.94	-1.89	0.62
Xuanshen	MOL000156	decaffeoylacteoside	462.5	6.33	-1.88	0.63
Xuanshen	MOL001660	p-MCA	178.2	31	0.75	0.05
Xuanshen	MOL001925	paeoniflorin_qt	318.35	68.18	-0.34	0.4
Xuanshen	MOL002222	sugiol	300.48	36.11	1.14	0.28
Xuanshen	MOL000223	caffeic acid	180.17	25.76	0.21	0.05
Xuanshen	MOL002295	cinnamic acid	148.17	19.68	0.91	0.03
Xuanshen	MOL002820	catapol_qt	200.21	44.69	-0.63	0.1
Xuanshen	MOL002922	5-(2-hydroxyethyl)-2-methoxyphenol	168.21	31.95	0.6	0.04
Xuanshen	MOL000346	succinic acid	118.1	29.62	-0.44	0.01
Xuanshen	MOL000356	lupeol	426.8	12.12	1.46	0.78
Xuanshen	MOL000357	Sitogluside	576.95	20.63	-0.14	0.62

Xuanshen	MOL000358	beta-sitosterol	414.79	36.91	1.32	0.75
Xuanshen	MOL000359	sitosterol	414.79	36.91	1.32	0.75
Xuanshen	MOL000360	FER	194.2	39.56	0.47	0.06
Xuanshen	MOL003735	aucubin	346.37	4.17	-1.83	0.33
Xuanshen	MOL000389	FERULIC ACID (CIS)	194.2	54.97	0.53	0.06
Xuanshen	MOL000429	Crystal VI	132.14	83.96	-0.88	0.02
Xuanshen	MOL000511	ursolic acid	456.78	16.77	0.67	0.75
Xuanshen	MOL006670	aucubigenin	184.21	101.77	-0.84	0.06
Xuanshen	MOL000675	oleic acid	282.52	33.13	1.17	0.14
Xuanshen	MOL007002	paeonioflorin	480.51	10.22	-1.69	0.79
Xuanshen	MOL007272	Homovanillyl alcohol	168.21	38.2	0.49	0.04
Xuanshen	MOL000748	HMF	126.12	45.07	0.05	0.02
Xuanshen	MOL007655	angroside C	784.84	6.48	-2.56	0.38
Xuanshen	MOL007656	scropolioside A	752.79	13	-2.24	0.35
Xuanshen	MOL007657	scropolioside A_qt	590.63	38.63	-0.99	0.77
Xuanshen	MOL007658	14-deoxy-12(R)-sulfoandrographolide	414.57	62.57	-0.38	0.42
Xuanshen	MOL007659	scropolioside D	722.76	36.62	-1.97	0.4

Xuanshen	MOL007660	scropolioside D_qt	560.6	33.17	-0.94	0.82
Xuanshen	MOL009040	harpagoside	494.54	15.53	-1.36	0.83
Xuanshen	MOL007662	harpagoside_qt	332.38	122.87	-0.17	0.32
Xuanshen	MOL007663	catapol	362.37	3.96	-1.83	0.44
Xuanshen	MOL004557	geniposide	388.41	14.64	-1.7	0.44
Xuanshen	MOL007665	geniposide_qt	226.25	4.58	-0.42	0.1
Xuanshen	MOL007666	5-(methylthio)valeronitrile	129.25	66.32	1.47	0.01
Xuanshen	MOL007667	6'-O-cinnamoylharpagirle	492.57	13.06	-1.26	0.82
Xuanshen	MOL007668	6'-O-cinnamoylharpagirle_qt 2	200.26	27.75	-0.5	0.08
Xuanshen	MOL007669	6-O-methylcatalpol	376.4	5.8	-1.29	0.48
Xuanshen	MOL007670	6-O-methylcatalpol_qt	214.24	60.28	-0.04	0.11
Xuanshen	MOL007671	harpagide	364.39	9.76	-2.13	0.36
Xuanshen	MOL007672	7-hydroxy-9-hydroxymethyl-3-oxo-	182.24	44.49	-0.54	0.06
Xuanshen	MOL007673	cistanoside D	652.71	12.91	-1.59	0.59
Xuanshen	MOL000875	Cedrol	222.41	16.23	1.35	0.12
Zhebeimu	MOL001004	pelargonidin	271.26	37.99	0.31	0.21
Zhebeimu	MOL003171	2,5-Dimethoxybenzoquinone	168.16	8.72	0.47	0.04

Zhebeimu	MOL000358	beta-sitosterol	414.79	36.91	1.32	0.75
Zhebeimu	MOL000365	syringaresinol	418.48	3.29	0.6	0.72
Zhebeimu	MOL004440	Peimisine	427.69	57.4	0.18	0.81
Zhebeimu	MOL004442	Pelargonidin-3,5-diglucoside	595.58	10.96	-2.87	0.76
Zhebeimu	MOL004443	Zhebeiresinol	280.3	58.72	0.53	0.19
Zhebeimu	MOL004444	Ziebeimine	413.71	64.25	0.81	0.7
Zhebeimu	MOL004445	Verticine	431.73	17.42	0.05	0.67
Zhebeimu	MOL004446	6-Methoxyl-2-acetyl-3-methyl-1,4-naphthoquinone-8-O-beta-D-glucopyranoside	422.42	33.31	-1.21	0.57
Zhebeimu	MOL004447	6-Methoxyl-2-acetyl-3-methyl-1,4-naphthoquinone-8-O-beta-D-glucopyranoside_qt	260.26	19.87	0.1	0.15
Zhebeimu	MOL004448	Solatubin	397.71	17.12	1.22	0.76
Zhebeimu	MOL004449	OSI-2040	623.88	14.65	0.13	0.61
Zhebeimu	MOL004450	Chaksine	450.66	65.63	-0.01	0.66
Zhebeimu	MOL004451	Peiminine	429.71	13.2	0.23	0.67
Zhebeimu	MOL004452	Peiminoside	593.89	2.25	-1.14	0.21
Zhebeimu	MOL004453	Peiminoside_qt	431.73	11.75	0.18	0.67
Shengmuli	tcmid_ID:23782	Aluminum	26.982	—	—	—
Shengmuli	tcmid_ID:23648	Calcium Sulphate	136.141	—	—	—

Shengmuli	tcmid_ID:25060	Calcium Phosphate	310.177	—	—	—
Shengmuli	tcmid_ID:23471	Silicon	28.086	—	—	—
Shengmuli	tcmid_ID:23378	Calcium Carbonate	100.087	—	—	—

Table S2 Detailed information about putative gene targets in XLW

XLW	Compound	Target Genes	Gene ID in UniProt
Xuanshen	sugiol	CHRM3	P20309
Xuanshen	sugiol	CHRM1	P11229
Xuanshen	sugiol	SCN5A	Q14524
Xuanshen	sugiol	CHRM5	P08912
Xuanshen	sugiol	PTGS2	P35354
Xuanshen	sugiol	CA2	P00918
Xuanshen	sugiol	CHRM4	P08173
Xuanshen	sugiol	OPRD1	P41143
Xuanshen	sugiol	ACHE	P22303
Xuanshen	sugiol	ADRA1A	P35348
Xuanshen	sugiol	CHRM2	P08172
Xuanshen	sugiol	ADRA1B	P35368
Xuanshen	sugiol	ADRB2	P07550
Xuanshen	sugiol	ADRA1D	P25100
Xuanshen	sugiol	DRD2	P14416
Xuanshen	sugiol	OPRM1	P35372
Xuanshen	sugiol	CHRNA7	P36544
Xuanshen	beta-sitosterol	PGR	P06401
Xuanshen	beta-sitosterol	NCOA2	Q15596
Xuanshen	beta-sitosterol	PTGS1	P23219
Xuanshen	beta-sitosterol	PTGS2	P35354
Xuanshen	beta-sitosterol	HSP90AB1	P08238
Xuanshen	beta-sitosterol	PIK3CG	P48736
Xuanshen	beta-sitosterol	KCNH2	Q12809
Xuanshen	beta-sitosterol	PRKACA	P17612
Xuanshen	beta-sitosterol	DRD1	P21728
Xuanshen	beta-sitosterol	CHRM3	P20309
Xuanshen	beta-sitosterol	CHRM1	P11229
Xuanshen	beta-sitosterol	SCN5A	Q14524
Xuanshen	beta-sitosterol	GABRA2	P47869
Xuanshen	beta-sitosterol	CHRM4	P08173
Xuanshen	beta-sitosterol	PDE3A	Q14432
Xuanshen	beta-sitosterol	HTR2A	P28223
Xuanshen	beta-sitosterol	GABRA5	P31644
Xuanshen	beta-sitosterol	ADRA1A	P35348
Xuanshen	beta-sitosterol	GABRA3	P34903
Xuanshen	beta-sitosterol	CHRM2	P08172

Xuanshen	beta-sitosterol	ADRA1B	P35368
Xuanshen	beta-sitosterol	ADRB2	P07550
Xuanshen	beta-sitosterol	CHRNA2	Q15822
Xuanshen	beta-sitosterol	SLC6A4	P31645
Xuanshen	beta-sitosterol	OPRM1	P35372
Xuanshen	beta-sitosterol	GABRA1	P14867
Xuanshen	beta-sitosterol	CHRNA7	P36544
Xuanshen	beta-sitosterol	CYP101A1	P00183
Xuanshen	beta-sitosterol	BCL2	P10415
Xuanshen	beta-sitosterol	BAX	Q07812
Xuanshen	beta-sitosterol	CASP9	P55211
Xuanshen	beta-sitosterol	JUN	P05412
Xuanshen	beta-sitosterol	CASP3	P42574
Xuanshen	beta-sitosterol	CASP8	Q14790
Xuanshen	beta-sitosterol	PRKCA	P17252
Xuanshen	beta-sitosterol	TGFB1	P01137
Xuanshen	beta-sitosterol	PON1	P27169
Xuanshen	beta-sitosterol	MAP2	P11137
Xuanshen	sitosterol	PGR	P06401
Xuanshen	sitosterol	NCOA2	Q15596
Xuanshen	sitosterol	NR3C2	P08235
Zhebeimu	beta-sitosterol	PGR	P06401
Zhebeimu	beta-sitosterol	NCOA2	Q15596
Zhebeimu	beta-sitosterol	PTGS1	P23219
Zhebeimu	beta-sitosterol	PTGS2	P35354
Zhebeimu	beta-sitosterol	HSP90AB1	P08238
Zhebeimu	beta-sitosterol	PIK3CG	P48736
Zhebeimu	beta-sitosterol	KCNH2	Q12809
Zhebeimu	beta-sitosterol	PRKACA	P17612
Zhebeimu	beta-sitosterol	DRD1	P21728
Zhebeimu	beta-sitosterol	CHRM3	P20309
Zhebeimu	beta-sitosterol	CHRM1	P11229
Zhebeimu	beta-sitosterol	SCN5A	Q14524
Zhebeimu	beta-sitosterol	GABRA2	P47869
Zhebeimu	beta-sitosterol	CHRM4	P08173
Zhebeimu	beta-sitosterol	PDE3A	Q14432
Zhebeimu	beta-sitosterol	HTR2A	P28223
Zhebeimu	beta-sitosterol	GABRA5	P31644
Zhebeimu	beta-sitosterol	ADRA1A	P35348
Zhebeimu	beta-sitosterol	GABRA3	P34903

Zhebeimu	beta-sitosterol	CHRM2	P08172
Zhebeimu	beta-sitosterol	ADRA1B	P35368
Zhebeimu	beta-sitosterol	ADRB2	P07550
Zhebeimu	beta-sitosterol	CHRNA2	Q15822
Zhebeimu	beta-sitosterol	SLC6A4	P31645
Zhebeimu	beta-sitosterol	OPRM1	P35372
Zhebeimu	beta-sitosterol	GABRA1	P14867
Zhebeimu	beta-sitosterol	CHRNA7	P36544
Zhebeimu	beta-sitosterol	CYP101A1	P00183
Zhebeimu	beta-sitosterol	BCL2	P10415
Zhebeimu	beta-sitosterol	BAX	Q07812
Zhebeimu	beta-sitosterol	CASP9	P55211
Zhebeimu	beta-sitosterol	JUN	P05412
Zhebeimu	beta-sitosterol	CASP3	P42574
Zhebeimu	beta-sitosterol	CASP8	Q14790
Zhebeimu	beta-sitosterol	PRKCA	P17252
Zhebeimu	beta-sitosterol	TGFB1	P01137
Zhebeimu	beta-sitosterol	PON1	P27169
Zhebeimu	beta-sitosterol	MAP2	P11137
Zhebeimu	Zhebeiresinol	PTGS1	P23219
Zhebeimu	Zhebeiresinol	SCN5A	Q14524
Zhebeimu	Zhebeiresinol	PTGS2	P35354
Zhebeimu	Zhebeiresinol	RXRA	P19793
Zhebeimu	Zhebeiresinol	PDE3A	Q14432
Zhebeimu	Zhebeiresinol	ADRB2	P07550
Zhebeimu	Zhebeiresinol	GABRA1	P14867
Zhebeimu	Zhebeiresinol	HSP90AB1	P08238
Shengmuli	Aluminum	TF	P02787
Shengmuli	Aluminum	ATP1A1	P05023
Shengmuli	Calcium Sulphate	CACNB1	Q02641
Shengmuli	Calcium Sulphate	CACNA2D1	P54289
Shengmuli	Calcium Sulphate	CACNA1S	Q13698
Shengmuli	Calcium Sulphate	CACNA1C	Q13936
Shengmuli	Calcium Sulphate	CACNB2	Q08289
Shengmuli	Calcium Sulphate	CACNG1	Q06432
Shengmuli	Calcium Sulphate	FOXL2	P58012
Shengmuli	Calcium Sulphate	DMTN	Q08495
Shengmuli	Calcium Sulphate	KCNMA1	Q12791
Shengmuli	Calcium Sulphate	P2RX2	Q9UBL9
Shengmuli	Calcium Sulphate	HTR2A	P28223

Shengmuli	Calcium Sulphate	P2RX3	P56373
Shengmuli	Calcium Sulphate	CHRNA3	P32297
Shengmuli	Calcium Sulphate	CHRNB2	P17787
Shengmuli	Calcium Sulphate	CHRNB4	P30926
Shengmuli	Calcium Phosphate	CACNB1	Q02641
Shengmuli	Calcium Phosphate	CACNA2D1	P54289
Shengmuli	Calcium Phosphate	SLC12A2	P55011
Shengmuli	Calcium Phosphate	SLC12A7	Q9Y666
Shengmuli	Calcium Phosphate	SLC12A5	Q9H2X9
Shengmuli	Calcium Phosphate	CACNA1S	Q13698
Shengmuli	Calcium Phosphate	CACNA1C	Q13936
Shengmuli	Calcium Phosphate	SLC12A6	Q9UHW9
Shengmuli	Calcium Phosphate	SLC12A1	Q13621
Shengmuli	Calcium Phosphate	CACNB2	Q08289
Shengmuli	Calcium Phosphate	SLC12A4	Q9UP95
Shengmuli	Calcium Phosphate	CACNG1	Q06432
Shengmuli	Silicon	ADRA1A	P35348
Shengmuli	Silicon	SLC18A2	Q05940
Shengmuli	Silicon	HTR1A	P08908
Shengmuli	Calcium Carbonate	FDPS	P14324

Table S3 Identified known therapeutic gene targets about UFs in DiGSeE database

PubMed ID	Gene	Event class	Event term	Searched disease	Evidence score
12821352	DNMT1	Gene expression	expression	leiomyoma uterine	0.825
12193211	GFAP	Gene expression	expression	leiomyoma uterine	0.821
16109840	CYP19A1	Binding	binding	leiomyoma uterine	0.804
10550310	HMGA2	Gene expression	expressed	leiomyoma uterine	0.799
15196440	PCNA	Gene expression	expression	leiomyoma uterine	0.784
12149410	ESR1	Gene expression	expressing	leiomyoma uterine	0.777
9159329	PCNA	Gene expression	expression	leiomyoma uterine	0.777
9263424	TMPO	Gene expression	expression	leiomyoma uterine	0.766
12065682	MYOZ2	Gene expression	expressed	leiomyoma uterine	0.766
10671823	NOS3	Gene expression	expression	leiomyoma uterine	0.762
10825369	PCNA	Gene expression	expression	leiomyoma uterine	0.761
10854234	CDKN2A	Gene expression	Expression	leiomyoma uterine	0.754
15051039	BCL2	Gene expression	expression	leiomyoma uterine	0.745
10611261	FOS	Gene expression	expression	leiomyoma uterine	0.742
16730007	COMT	Gene expression	expression	leiomyoma uterine	0.736
1516585	KRAS	Gene expression	expression	leiomyoma uterine	0.735
17001917	MME	Gene expression	expressed	leiomyoma uterine	0.733
16973256	TP53	Gene expression	expression	leiomyoma uterine	0.733
12920818	SLC4A1	Gene expression	expression	leiomyoma uterine	0.73
2208134	IGF2	Gene expression	expressed	leiomyoma uterine	0.73
10202672	INHA	Gene expression	expression	leiomyoma uterine	0.729
10967169	NOS3	Gene expression	expression	leiomyoma uterine	0.728
12920806	EGFR	Gene expression	expressions	leiomyoma uterine	0.728
12133359	KIT	Gene expression	expression	leiomyoma uterine	0.727
15139000	DPT	Gene expression	expression	leiomyoma uterine	0.726
15051038	MMP2	Gene expression	expression	leiomyoma uterine	0.723
10230352	LGALS3	Gene expression	expression	leiomyoma uterine	0.722
7775635	ESR1	Gene expression	expression	leiomyoma uterine	0.72
11957148	PDAP1	Gene expression	expression	leiomyoma uterine	0.718
10956556	ADRB2	Gene expression	expression	leiomyoma uterine	0.718
10682457	EGF	Gene expression	expression	leiomyoma uterine	0.717
10830768	TP53	Gene expression	expression	leiomyoma uterine	0.711
12097287	TSC2	Gene expression	expression	leiomyoma uterine	0.71

10426571	PTHLH	Gene expression	expression	leiomyoma uterine	0.71
17300669	NES	Gene expression	expression	leiomyoma uterine	0.71
10394541	BCL2	Gene expression	expression	leiomyoma uterine	0.709
10398424	HMGA2	Gene expression	expression	leiomyoma uterine	0.708
12874903	DES	Gene expression	expression	leiomyoma uterine	0.706
14981145	WISP2	Gene expression	expression	leiomyoma uterine	0.705
10545803	PCNA	Gene expression	expression	leiomyoma uterine	0.704
10698042	IGFBP7	Gene expression	expression	leiomyoma uterine	0.704
16146740	CYP19A1	Gene expression	expression	leiomyoma uterine	0.704
12111198	RBP1	Gene expression	expression	leiomyoma uterine	0.703
10682471	PGR	Gene expression	expression	leiomyoma uterine	0.703
16697947	TGFB3	Gene expression	expression	leiomyoma uterine	0.703
9216720	HMGA2	Gene expression	expression	leiomyoma uterine	0.702
17341559	CYP19A1	Gene expression	expression	leiomyoma uterine	0.702
1333524	TP53	Gene expression	expression	leiomyoma uterine	0.7
17332316	HMGA2	Gene expression	expressed	leiomyoma uterine	0.698
2670616	VIM	Gene expression	expression	leiomyoma uterine	0.698
17173903	PTN	Gene expression	expressed	leiomyoma uterine	0.692
10086344	KIT	Gene expression	expression	leiomyoma uterine	0.691
15059325	KIT	Gene expression	expression	leiomyoma uterine	0.689
10362815	COL4A5	Transcription	transcription	leiomyoma uterine	0.688
10850463	VIPR2	Gene expression	express	leiomyoma uterine	0.687
15322223	S100A11	Gene expression	expression	leiomyoma uterine	0.68
10371341	CDK2	Gene expression	expression	leiomyoma uterine	0.678
10559661	CYP19A1	Gene expression	expressed	leiomyoma uterine	0.673
12111201	BAX	Gene expression	expression	leiomyoma uterine	0.673
15231681	EGR1	Gene expression	expression	leiomyoma uterine	0.672
9290461	PCNA	Gene expression	expression	leiomyoma uterine	0.671
11193811	VIPR2	Gene expression	express	leiomyoma uterine	0.668
10601839	AR	Gene expression	expression	leiomyoma uterine	0.668
19729795	ESR1	Mutation	T/C	leiomyoma uterine	0.653
10208423	LTA	Gene expression	expressing	leiomyoma uterine	0.651
10914728	VEGFA	Gene expression	expressed	leiomyoma uterine	0.65
10785229	TGFB3	Gene expression	expression	leiomyoma uterine	0.648
15066464	ESR1	Gene expression	expression	leiomyoma uterine	0.646
11867266	BCL2	Gene expression	expression	leiomyoma uterine	0.644

9253340	FOS	Gene expression	expression	leiomyoma uterine	0.639
17316415	KIT	Gene expression	expressing	leiomyoma uterine	0.639
10508223	LCN2	Gene expression	expression	leiomyoma uterine	0.631
12909487	FZD2	Gene expression	expression	leiomyoma uterine	0.631
11870232	ZKSCAN7	Gene expression	expression	leiomyoma uterine	0.626
10450179	LCN2	Gene expression	expression	leiomyoma uterine	0.618
17276435	TFPI	Gene expression	expression	leiomyoma uterine	0.617
15193492	CD36	Gene expression	expression	leiomyoma uterine	0.614
19639498	ESR2	Mutation	rs3020450	leiomyoma uterine	0.593
25792652	PON1	Mutation	Q192R	leiomyoma uterine	0.593
15033949	ESR1	Gene expression	expression	leiomyoma uterine	0.59
16681772	TP53	Gene expression	expressed	leiomyoma uterine	0.589
18182398	CCND1	Mutation	G870A	leiomyoma uterine	0.584
20013148	XRCC1	Mutation	Arg280His	leiomyoma uterine	0.584
26261478	HSD17B1	Mutation	rs605059	leiomyoma uterine	0.584
26064254	CYP1A1	Mutation	Ile462Val	leiomyoma uterine	0.584
9294852	FGFR1	Gene expression	expression	leiomyoma uterine	0.582
10352391	PRL	Gene expression	production	leiomyoma uterine	0.581
17296196	GNRH2	Gene expression	expression	leiomyoma uterine	0.581
12201218	PTH	Gene expression	expressed	leiomyoma uterine	0.574
17268837	KIT	Gene expression	expressed	leiomyoma uterine	0.574
26254358	XRCC3	Mutation	rs45603942	leiomyoma uterine	0.573
17170071	CRABP2	Transcription	transcription	leiomyoma uterine	0.573
11872215	OVGP1	Gene expression	expression	leiomyoma uterine	0.571
17336977	CYP19A1	Gene expression	expression	leiomyoma uterine	0.567
11201279	AKR1C4	Gene expression	expression	leiomyoma uterine	0.561
26113603	RGS7	Mutation	rs78220092	leiomyoma uterine	0.558
17636223	COMT	Mutation	Val158Met	leiomyoma uterine	0.558
16638593	MMP1	Mutation	-1562 C/T	leiomyoma uterine	0.558
10633228	GJA1	Gene expression	Expression	leiomyoma uterine	0.558
1309926	PDGFRB	Gene expression	expression	leiomyoma uterine	0.556
15860490	VEGFA	Gene expression	expression	leiomyoma uterine	0.552
1310202	HTLVR	Binding	binding	leiomyoma uterine	0.549
17908262	FH	Mutation	R58P	leiomyoma uterine	0.547
12620485	CYP19A1	Gene expression	expression	leiomyoma uterine	0.545
20427420	TSHR	Mutation	M453T	leiomyoma uterine	0.54

17220163	PCNA	Gene expression	expression	leiomyoma uterine	0.54
10027613	OXT	Localization	localization	leiomyoma uterine	0.54
16160478	ALK	Gene expression	expression	leiomyoma uterine	0.538
10860276	PGR	Gene expression	expression	leiomyoma uterine	0.535
15663510	FH	Mutation	ubstitution at nucleo	leiomyoma uterine	0.535
17186536	TP53	Gene expression	expression	leiomyoma uterine	0.532
16962107	BCL2	Gene expression	expression	leiomyoma uterine	0.532
15949892	FH	Gene expression	expression	leiomyoma uterine	0.532
18164802	CASP3	Gene expression	expression	leiomyoma uterine	0.517
12922981	MYC	Gene expression	expression	leiomyoma uterine	0.516
2112150	PRL	Localization	secretion	leiomyoma uterine	0.515
2768133	DES	Gene expression	expression	leiomyoma uterine	0.514
16935316	ESR1	Mutation	rs17847075	leiomyoma uterine	0.512
17011556	ESR2	Localization	localization	leiomyoma uterine	0.511
16778173	RAD51B	Gene expression	expression	leiomyoma uterine	0.507
10029085	PLAG1	Gene expression	expression	leiomyoma uterine	0.507
12090595	DES	Gene expression	expression	leiomyoma uterine	0.503
17164439	SPHK1	Positive regulation	increased	leiomyoma uterine	0.501
15228428	CCND1	Gene expression	expression	leiomyoma uterine	0.491
10611187	PGR	Gene expression	expression	leiomyoma uterine	0.486
10620447	ESR1	Transcription	transcription	leiomyoma uterine	0.483
14561812	GAS6	Gene expression	expression	leiomyoma uterine	0.478
15032287	CDH1	Gene expression	expression	leiomyoma uterine	0.477
17976709	GNRH1	Localization	Releasing	leiomyoma uterine	0.476
17278201	DES	Gene expression	expression	leiomyoma uterine	0.474
16959847	SPHK1	Gene expression	expression	leiomyoma uterine	0.472
15164305	ESR1	Binding	bind	leiomyoma uterine	0.466
2797644	IGF1R	Binding	binding	leiomyoma uterine	0.466
17021135	KIT	Gene expression	expressed	leiomyoma uterine	0.457
18231572	SHC1	Transcription	overexpressed	leiomyoma uterine	0.452
21143902	SELENBP1	Gene expression	expression	leiomyoma uterine	0.452
12624519	ERBB2	Gene expression	expression	leiomyoma uterine	0.447
2116610	IGF2	Localization	secretion	leiomyoma uterine	0.446
19715484	ESR2	Mutation	rs1256049	leiomyoma uterine	0.446
16595228	COMT	Mutation	A 34T-->C	leiomyoma uterine	0.446
15285309	ESR1	Gene expression	expression	leiomyoma uterine	0.443

17407572	ANXA1	Gene expression	expression	leiomyoma uterine	0.44
22546946	HSD17B1	Mutation	rs605059	leiomyoma uterine	0.437
20559649	CYP1B1	Mutation	Leu 432Val	leiomyoma uterine	0.437
18763031	CYP1A1	Mutation	Ile462Val	leiomyoma uterine	0.437
23238870	COMT	Mutation	Val158Met	leiomyoma uterine	0.437
25511444	MDM2	Mutation	T309G	leiomyoma uterine	0.437
26692154	TP53	Mutation	Arg72Pro	leiomyoma uterine	0.437
16051682	PPARG	Positive regulation	roliferation-activate	leiomyoma uterine	0.436
17343855	CCL2	Gene expression	production	leiomyoma uterine	0.434
15604208	CDKN1C	Gene expression	expression	leiomyoma uterine	0.431
10566662	BMP6	Gene expression	express	leiomyoma uterine	0.426
12095500	MAP3K12	Gene expression	expressed	leiomyoma uterine	0.423
9299534	PRL	Gene expression	expression	leiomyoma uterine	0.42
9159347	MDM2	Gene expression	overexpression	leiomyoma uterine	0.416
11869553	CCND1	Gene expression	Overexpression	leiomyoma uterine	0.413
21138652	IL4	Mutation	-590 C/T	leiomyoma uterine	0.411
26075073	CYP1B1	Mutation	rs1800440	leiomyoma uterine	0.411
24777039	CYP1A1	Mutation	Ala119Ser	leiomyoma uterine	0.408
16972175	AKR1B10	Mutation	c.976C > T	leiomyoma uterine	0.408
9157826	VIM	Gene expression	coexpression	leiomyoma uterine	0.407
11938733	ESR1	Gene expression	expression	leiomyoma uterine	0.406
2778330	TGFB1	Positive regulation	increased	leiomyoma uterine	0.406
18569028	ATP8A2	Gene expression	expression	leiomyoma uterine	0.404
17313836	PML	Gene expression	expression	leiomyoma uterine	0.4
18601603	CYP17A1	Mutation	rs743572	leiomyoma uterine	0.399
19614627	IL1B	Mutation	-511 C<T	leiomyoma uterine	0.399
26417400	SORCS2	Mutation	rs2285789	leiomyoma uterine	0.399
26482777	CYP1B1	Mutation	Leu432Val	leiomyoma uterine	0.399
26704829	MME	Mutation	P504S	leiomyoma uterine	0.399
18580309	MKI67	Gene expression	expression	leiomyoma uterine	0.392
10784614	ZNF382	Binding	bound	leiomyoma uterine	0.388
10774817	PGR	Gene expression	expression	leiomyoma uterine	0.385
15589581	CDKN2A	Gene expression	expression	leiomyoma uterine	0.383
12413981	HOXA10	Gene expression	express	leiomyoma uterine	0.383
15086838	NCAM1	Gene expression	expression	leiomyoma uterine	0.383
17036392	VEGFA	Gene expression	Expression	leiomyoma uterine	0.383

15724016	FH	Mutation	G354R	leiomyoma uterine	0.38
25985877	FH	Mutation	p.(His318Leu)	leiomyoma uterine	0.38
19448595	PDGFRA	Mutation	1836_1850del	leiomyoma uterine	0.38
16757530	FH	Mutation	N64T	leiomyoma uterine	0.38
18645709	CYP17A1	Mutation	rs1042386	leiomyoma uterine	0.374
17270242	DES	Gene expression	negative	leiomyoma uterine	0.37
26702771	FBLN1	Gene expression	expression	leiomyoma uterine	0.362
17531625	BCL2	Gene expression	expression	leiomyoma uterine	0.359
16720624	VEGFA	Positive regulation	progesterone-induce	leiomyoma uterine	0.348
10758788	HESX1	Localization	release	leiomyoma uterine	0.339
18956301	SHBG	Regulation	changes	leiomyoma uterine	0.336
23551056	PDCD6	Mutation	rs3756712	leiomyoma uterine	0.334
26396919	MED12	Mutation	c.122-123ins15	leiomyoma uterine	0.334
9495195	HMGA2	Gene expression	overexpressed	leiomyoma uterine	0.332
15536154	PCNA	Gene expression	expression	leiomyoma uterine	0.33
21453057	ND3	Mutation	G3316A	leiomyoma uterine	0.329
23612258	FH	Mutation	.1431_1433dupAA	leiomyoma uterine	0.329
15922007	IGF1	Gene expression	expression	leiomyoma uterine	0.328
10968348	HESX1	Localization	release	leiomyoma uterine	0.322
17380299	PTGS2	Regulation	effect	leiomyoma uterine	0.32
10519970	EDNRB	Binding	binds	leiomyoma uterine	0.319
7882774	TP53	Gene expression	overexpression	leiomyoma uterine	0.317
12937923	IRS1	Transcription	levels	leiomyoma uterine	0.316
10972520	PLOD1	Localization	secretion	leiomyoma uterine	0.314
10680889	DES	Gene expression	coexpression	leiomyoma uterine	0.314
1532397	MUC16	Gene expression	producing	leiomyoma uterine	0.309
26193636	MED12	Gene expression	expression	leiomyoma uterine	0.308
26416628	PPP4C	Gene expression	expression	leiomyoma uterine	0.308
16892079	HK2	Positive regulation	induce	leiomyoma uterine	0.308
15941862	GRPR	Gene expression	expression	leiomyoma uterine	0.306
11786376	IRS1	Phosphorylation	phosphorylation	leiomyoma uterine	0.305
10065869	PSMD9	Positive regulation	induction	leiomyoma uterine	0.304
15572421	PCNA	Gene expression	expression	leiomyoma uterine	0.302
26261614	CDKN2A	Gene expression	expression	leiomyoma uterine	0.3
10862042	HMGA2	Localization	localized	leiomyoma uterine	0.294
26691377	CD34	Gene expression	expressing	leiomyoma uterine	0.289

2112489	EREG	Positive regulation	increase	leiomyoma uterine	0.28
14990970	HV1S	Gene expression	using	leiomyoma uterine	0.279
20171624	CYP17A1	Mutation	rs743572	leiomyoma uterine	0.275
26236334	TNRC6B	Mutation	rs739182	leiomyoma uterine	0.275
22853432	MMP3	Mutation	rs3025058	leiomyoma uterine	0.275
16835796	PPIG	Binding	association	leiomyoma uterine	0.273
1730337	CGA	Gene expression	production	leiomyoma uterine	0.272
15140868	EGF	Positive regulation	up-regulates	leiomyoma uterine	0.27
16443508	COMT	Mutation	Val158Met	leiomyoma uterine	0.268
26715264	PGR	Mutation	+331G/A	leiomyoma uterine	0.268
18555241	MMRN1	Gene expression	production	leiomyoma uterine	0.265
18089606	IGF1	Gene expression	expression	leiomyoma uterine	0.264
26405405	PIP	Gene expression	Expression	leiomyoma uterine	0.263
10383698	TP53	Gene expression	overexpression	leiomyoma uterine	0.261
10362625	EDEM1	Positive regulation	increased	leiomyoma uterine	0.256
14695314	FH	Mutation	Ala196Thr	leiomyoma uterine	0.253
21506659	ESR1	Mutation	-397T > C	leiomyoma uterine	0.253
23479081	POLR2E	Mutation	rs34917480	leiomyoma uterine	0.253
26383637	MED12	Mutation	L1224F	leiomyoma uterine	0.253
20560959	FH	Mutation	p.Ala274Val	leiomyoma uterine	0.253
12115525	CUX1	Positive regulation	increase	leiomyoma uterine	0.253
10764475	GJA1	Localization	localization	leiomyoma uterine	0.25
15355882	MAPK1	Positive regulation	activation	leiomyoma uterine	0.247
26552702	HOXA11	Gene expression	expression	leiomyoma uterine	0.246
16330948	CDKN2A	Negative regulation	panel	leiomyoma uterine	0.242
18208801	ACTC1	Binding	immunoreactivity	leiomyoma uterine	0.242
12923133	SERPINA7	Regulation	effect	leiomyoma uterine	0.236
11921278	HMGA2	Gene expression	expression	leiomyoma uterine	0.233
12900513	COL4A2	Positive regulation	increased	leiomyoma uterine	0.23
15518168	CGA	Gene expression	present	leiomyoma uterine	0.227
10792849	FSHR	Localization	secretion	leiomyoma uterine	0.227
19088479	HIF1A	Gene expression	expression	leiomyoma uterine	0.227
15979354	NCOA2	Gene expression	expression	leiomyoma uterine	0.226
7902208	IGF2	Mutation	C-->T	leiomyoma uterine	0.225
10574596	TP53	Mutation	rtate substitution of	leiomyoma uterine	0.225
19047911	TP53	Mutation	don 559 (GTT-->GA	leiomyoma uterine	0.225

18686281	PDGFRA	Mutation	Delta845-848	leiomyoma uterine	0.225
19013437	ESR1	Mutation	T47D	leiomyoma uterine	0.225
17356170	SGK1	Mutation	T47D	leiomyoma uterine	0.225
24684806	FH	Mutation	c.1021G > A	leiomyoma uterine	0.225
22223266	MED12	Mutation	G>A	leiomyoma uterine	0.225
15837988	GSN	Mutation	V559A	leiomyoma uterine	0.225
15761418	FH	Mutation	905-1G>A	leiomyoma uterine	0.225
16950754	AR	Mutation	R831X	leiomyoma uterine	0.225
16309500	FH	Mutation	T287P	leiomyoma uterine	0.225
15937070	FH	Mutation	R190H	leiomyoma uterine	0.225
14606094	KIT	Mutation	579ins12	leiomyoma uterine	0.225
12772087	FH	Mutation	R190H	leiomyoma uterine	0.225
22764886	FH	Mutation	c.584T>C	leiomyoma uterine	0.225
17960613	FH	Mutation	R190H	leiomyoma uterine	0.225
10461005	PRL	Gene expression	expression	leiomyoma uterine	0.225
1531577	GNRHR	Regulation	role	leiomyoma uterine	0.223
14566855	TSC2	Negative regulation	defect	leiomyoma uterine	0.222
17652152	DDIT3	Negative regulation	modulating	leiomyoma uterine	0.22
17697600	CCL5	Gene expression	expression	leiomyoma uterine	0.218
16613890	EGF	Gene expression	expression	leiomyoma uterine	0.218
2764227	PGR	Gene expression	expressed	leiomyoma uterine	0.216
11704174	STS	Localization	localization	leiomyoma uterine	0.213
18203783	MKI67	Gene expression	expressions	leiomyoma uterine	0.213
2126737	GNRH1	Localization	release	leiomyoma uterine	0.212
17506483	CGA	Gene expression	found	leiomyoma uterine	0.212
17970643	GNRHR	Gene expression	expression	leiomyoma uterine	0.212
16113040	GNRH1	Gene expression	production	leiomyoma uterine	0.21
18182067	MIR18A	Gene expression	expression	leiomyoma uterine	0.209
2110713	ESR1	Negative regulation	suppresses	leiomyoma uterine	0.209
15586379	THBS1	Gene expression	expression	leiomyoma uterine	0.208
15043312	SUZ12	Transcription	transcripts	leiomyoma uterine	0.207
15972578	WNT5B	Gene expression	Overexpression	leiomyoma uterine	0.206
17636224	DES	Gene expression	exposed	leiomyoma uterine	0.206
17362977	EDEM1	Gene expression	found	leiomyoma uterine	0.206
12145978	FSHR	Localization	secretion	leiomyoma uterine	0.206
17290603	MUC16	Positive regulation	elevated	leiomyoma uterine	0.206

17022307	PLOD1	Localization	release	leiomyoma uterine	0.205
7779084	TERF1	Negative regulation	reduction	leiomyoma uterine	0.204
15042000	TP53	Positive regulation	induction	leiomyoma uterine	0.203
10782888	DES	Localization	localized	leiomyoma uterine	0.201
17718906	EGR3	Gene expression	expression	leiomyoma uterine	0.2
16623782	PGR	Gene expression	expressed	leiomyoma uterine	0.198
12218210	IL1R1	Gene expression	Expression	leiomyoma uterine	0.198
16831287	CD34	Gene expression	expression	leiomyoma uterine	0.196
15112348	KIT	Gene expression	expression	leiomyoma uterine	0.196
18617617	IL8	Gene expression	expression	leiomyoma uterine	0.187
17072826	TDGF1	Gene expression	Overexpression	leiomyoma uterine	0.187
17222838	TGFB3	Gene expression	underexpression	leiomyoma uterine	0.186
1392332	ESR1	Positive regulation	negative	leiomyoma uterine	0.184
12125998	PGR	Gene expression	positive	leiomyoma uterine	0.183
12649180	ESR1	Negative regulation	Inhibition	leiomyoma uterine	0.181
11865300	FH	Negative regulation	reduced	leiomyoma uterine	0.181
26457356	FH	Localization	Detection	leiomyoma uterine	0.181
15967901	HRAS	Positive regulation	positive	leiomyoma uterine	0.18
17439725	SRC	Binding	associated	leiomyoma uterine	0.176
17306671	PIK3R1	Positive regulation	Requirements	leiomyoma uterine	0.176
26783463	CYP19A1	Negative regulation	inhibitors	leiomyoma uterine	0.176
11788889	GNRH1	Regulation	effect	leiomyoma uterine	0.175
15232741	ENO2	Localization	locations	leiomyoma uterine	0.174
7805461	HRAS	Gene expression	lower-expression	leiomyoma uterine	0.173
12620453	CYP19A1	Negative regulation	inhibitor	leiomyoma uterine	0.173
18539894	ESR1	Gene expression	expression	leiomyoma uterine	0.173
17361005	MAPK1	Gene expression	expression	leiomyoma uterine	0.171
15255840	FSHR	Positive regulation	increments	leiomyoma uterine	0.171
12215337	PECAM1	Gene expression	Expression	leiomyoma uterine	0.171
25922306	TET1	Positive regulation	elevated	leiomyoma uterine	0.169
15958792	HMGA1	Regulation	affecting	leiomyoma uterine	0.169
12057733	GNRH1	Regulation	effect	leiomyoma uterine	0.167
16607449	EDEM1	Gene expression	expression	leiomyoma uterine	0.167
10329038	TP53	Protein catabolism	degradation	leiomyoma uterine	0.166
17724803	TEK	Gene expression	expression	leiomyoma uterine	0.166
15938828	SMN1	Gene expression	expression	leiomyoma uterine	0.166

10593388	CYP2C9	Gene expression	expression	leiomyoma uterine	0.166
17602685	ESR1	Gene expression	expression	leiomyoma uterine	0.163
17415642	RAPH1	Gene expression	transformation	leiomyoma uterine	0.162
15987702	HIF1A	Gene expression	over-expression	leiomyoma uterine	0.162
18089612	THBS2	Gene expression	expression	leiomyoma uterine	0.16
14501815	FGF2	Gene expression	expressing	leiomyoma uterine	0.158
16122189	CP	Gene expression	synthesize	leiomyoma uterine	0.156
16429426	RPL22P1	Gene expression	positive	leiomyoma uterine	0.156
17253165	KIT	Gene expression	expressions	leiomyoma uterine	0.155
15448018	TCEB1	Binding	complex	leiomyoma uterine	0.15
19091325	PGR	Gene expression	expressed	leiomyoma uterine	0.149
18559518	EPAS1	Gene expression	expressed	leiomyoma uterine	0.148
17237842	GNRH1	Negative regulation	downregulation	leiomyoma uterine	0.144
26534934	EXOSC10	Gene expression	expression	leiomyoma uterine	0.144
17360930	FBN1	Gene expression	expression	leiomyoma uterine	0.143
15623465	BAX	Gene expression	expression	leiomyoma uterine	0.141
14748838	ITGB1	Gene expression	expression	leiomyoma uterine	0.141
17020966	FZD4	Binding	recognized	leiomyoma uterine	0.14
12942572	CAPN6	Gene expression	overexpressed	leiomyoma uterine	0.138
16009172	ANGPT2	Mutation	G/A	leiomyoma uterine	0.138
19189127	KIT	Mutation	S566R	leiomyoma uterine	0.138
20043998	SRC	Mutation	T47D	leiomyoma uterine	0.138
9740667	BCR	Mutation	S1461E	leiomyoma uterine	0.138
20628236	FH	Mutation	p.Gly232AspfsX24	leiomyoma uterine	0.138
21119048	CDK2	Mutation	T47D	leiomyoma uterine	0.138
23607964	PGR	Mutation	T47D	leiomyoma uterine	0.138
17270241	FH	Mutation	N330S	leiomyoma uterine	0.138
24525513	NF2	Mutation	c.362+1G>A	leiomyoma uterine	0.138
16477632	FH	Mutation	K424R	leiomyoma uterine	0.138
16639410	FH	Mutation	435insAAA	leiomyoma uterine	0.138
22867999	FH	Mutation	p.His192Asp	leiomyoma uterine	0.138
15073606	TP53	Mutation	E336X	leiomyoma uterine	0.138
18729310	MEN1	Mutation	D350V	leiomyoma uterine	0.138
14681320	INHA	Gene expression	expression	leiomyoma uterine	0.137
14707872	CCND1	Gene expression	Overexpression	leiomyoma uterine	0.137
17635941	ESR1	Positive regulation	induced	leiomyoma uterine	0.134

15171320	ESR1	Gene expression	expressed	leiomyoma uterine	0.133
16172143	RXRA	Gene expression	expressions	leiomyoma uterine	0.131
15044692	PLOD1	Negative regulation	decreased	leiomyoma uterine	0.13
17211469	FH	Negative regulation	diminished/absent	leiomyoma uterine	0.13
10235477	DDT	Negative regulation	effect	leiomyoma uterine	0.127
16952848	KIT	Gene expression	express	leiomyoma uterine	0.125
11979371	CD34	Gene expression	positive	leiomyoma uterine	0.125
15249743	ADIPOQ	Negative regulation	decreased	leiomyoma uterine	0.124
18565690	MME	Gene expression	expression	leiomyoma uterine	0.124
11955330	KIT	Positive regulation	negative	leiomyoma uterine	0.123
10228907	MUC16	Positive regulation	elevated	leiomyoma uterine	0.12
17646097	EDEM1	Gene expression	expression	leiomyoma uterine	0.119
17291269	SMUG1	Gene expression	using	leiomyoma uterine	0.119
12669243	SCLC1	Positive regulation	generate	leiomyoma uterine	0.119
12839670	KIT	Gene expression	negative	leiomyoma uterine	0.117
2189250	VIM	Binding	positivity	leiomyoma uterine	0.117
16996566	DES	Positive regulation	negative	leiomyoma uterine	0.116
1587488	B3GAT1	Gene expression	positive	leiomyoma uterine	0.116
12801270	NBR1	Positive regulation	elevated	leiomyoma uterine	0.113
10685334	PLOD1	Localization	secretion	leiomyoma uterine	0.111
15335251	MME	Positive regulation	negative	leiomyoma uterine	0.108
12823048	ACTC1	Binding	negative	leiomyoma uterine	0.108
16563231	TP63	Gene expression	leiomyosarcomas	leiomyoma uterine	0.108
16860797	PPY	Binding	associated	leiomyoma uterine	0.107
2126024	PLOD1	Negative regulation	suppressed	leiomyoma uterine	0.106
10726441	PLOD1	Positive regulation	levels	leiomyoma uterine	0.106
10559617	KLK3	Regulation	influence	leiomyoma uterine	0.106
12144683	LDHC	Positive regulation	elevated	leiomyoma uterine	0.105
26468330	HMGA2	Gene expression	coexist	leiomyoma uterine	0.105
18248652	TGFB3	Gene expression	expression	leiomyoma uterine	0.104
15374708	MAP3K5	Binding	interest	leiomyoma uterine	0.103
16641167	CYP19A1	Regulation	effect	leiomyoma uterine	0.103
17010149	PLAUR	Negative regulation	decreased	leiomyoma uterine	0.101
10051989	DES	Positive regulation	negative	leiomyoma uterine	0.101
16954695	TKT	Gene expression	transfected	leiomyoma uterine	0.1
12893208	KIT	Gene expression	expression	leiomyoma uterine	0.099

10499074	COL4A2	Gene expression	overexpression	leiomyoma uterine	0.098
10686944	HMGA2	Regulation	affected	leiomyoma uterine	0.098
16997932	PGR	Regulation	affected	leiomyoma uterine	0.095
25735342	ACE	Regulation	influence	leiomyoma uterine	0.094
15760950	XRCC1	Binding	associated	leiomyoma uterine	0.094
10534170	DES	Positive regulation	negative	leiomyoma uterine	0.094
16365012	RRS1	Positive regulation	increases	leiomyoma uterine	0.093
12385821	PGR	Gene expression	expression	leiomyoma uterine	0.092
16363371	EGF	Binding	bind	leiomyoma uterine	0.092
15823826	MKI67	Gene expression	expression	leiomyoma uterine	0.092
25824050	CYP19A1	Negative regulation	inhibitors	leiomyoma uterine	0.091
7804565	LPL	Gene expression	assessed	leiomyoma uterine	0.09
16810068	ACTC1	Positive regulation	negative	leiomyoma uterine	0.09
19108974	NR3C2	Binding	intensity	leiomyoma uterine	0.09
17605689	NR3C2	Gene expression	produced	leiomyoma uterine	0.089
16390776	FSHR	Gene expression	expression	leiomyoma uterine	0.089
16649417	CYP19A1	Regulation	use	leiomyoma uterine	0.089
19706309	GNRH1	Negative regulation	inhibits	leiomyoma uterine	0.088
18216291	LCN2	Negative regulation	inhibitors	leiomyoma uterine	0.087
17342290	PLOD2	Binding	occurred	leiomyoma uterine	0.087
19122463	EBAG9	Regulation	alterations	leiomyoma uterine	0.086
15618963	HMGA2	Gene expression	expression	leiomyoma uterine	0.086
15587289	KIT	Gene expression	expression	leiomyoma uterine	0.086
10796723	GNRH1	Gene expression	improved	leiomyoma uterine	0.085
10383697	ENO2	Binding	negativity	leiomyoma uterine	0.085
15665689	ADC	Localization	embolization	leiomyoma uterine	0.084
15205403	LCK	Negative regulation	inhibitors	leiomyoma uterine	0.083
18226017	S100B	Gene expression	negative	leiomyoma uterine	0.083
26458617	PGR	Gene expression	negative	leiomyoma uterine	0.083
2140991	SNORA62	Negative regulation	decrease	leiomyoma uterine	0.081
9160309	DES	Gene expression	negative	leiomyoma uterine	0.08
12024318	S100B	Gene expression	positive	leiomyoma uterine	0.08
14501826	VIM	Regulation	immunoreactive	leiomyoma uterine	0.08
12411015	VEGFA	Gene expression	expression	leiomyoma uterine	0.08
15039185	SERPINB1	Regulation	calculated	leiomyoma uterine	0.079
15083381	HSD17B7	Gene expression	overexpressed	leiomyoma uterine	0.079

15213600	MME	Gene expression	positive	leiomyoma uterine	0.079
10397250	ESR1	Regulation	modulate	leiomyoma uterine	0.079
9169041	HMGA2	Gene expression	detected	leiomyoma uterine	0.078
26037152	MED12	Regulation	regulator	leiomyoma uterine	0.077
10856486	ESR2	Positive regulation	Expression	leiomyoma uterine	0.076
1710946	S100B	Positive regulation	negative	leiomyoma uterine	0.074
15307213	ACTC1	Gene expression	detect	leiomyoma uterine	0.073
18154577	CD34	Gene expression	expressed	leiomyoma uterine	0.073
12494390	PCYT1A	Localization	localization	leiomyoma uterine	0.073
15571618	MME	Gene expression	expression	leiomyoma uterine	0.073
16462152	CDKN2A	Gene expression	expression	leiomyoma uterine	0.073
1296036	VIM	Positive regulation	positive	leiomyoma uterine	0.067
18555231	HOXA10	Gene expression	expression	leiomyoma uterine	0.067
2070087	VEGFA	Regulation	influence	leiomyoma uterine	0.065
1317649	GNRH1	Positive regulation	promoted	leiomyoma uterine	0.065
10502446	RAPH1	Gene expression	transformation	leiomyoma uterine	0.064
16802186	AGRP	Negative regulation	reduces	leiomyoma uterine	0.064
10679920	HMGA2	Regulation	affected	leiomyoma uterine	0.063
12296380	PLA2G2A	Positive regulation	up-regulation	leiomyoma uterine	0.063
12135868	FSHR	Positive regulation	null	leiomyoma uterine	0.062
17285575	None	Negative regulation	deletion	leiomyoma uterine	0.062
1283316	DDIT3	Positive regulation	assumed	leiomyoma uterine	0.062
1287735	NR3C2	Gene expression	appearance	leiomyoma uterine	0.061
16443507	PLOD1	Positive regulation	activating	leiomyoma uterine	0.061
10565301	RAD51B	Regulation	interrupted	leiomyoma uterine	0.059
18156978	CDKN2A	Gene expression	overexpression	leiomyoma uterine	0.058
1539451	DES	Gene expression	positive	leiomyoma uterine	0.058
16408199	EGF	Gene expression	positive	leiomyoma uterine	0.058
12207602	CR2	Transcription	demonstrated	leiomyoma uterine	0.057
18177646	XRCC4	Binding	associated	leiomyoma uterine	0.056
16209296	BCL2	Gene expression	studied	leiomyoma uterine	0.056
17418408	HIF1A	Gene expression	overexpressed	leiomyoma uterine	0.054
18214799	TNF	Gene expression	synthesis	leiomyoma uterine	0.052
12963511	FSHR	Gene expression	levels	leiomyoma uterine	0.052
17690535	NR3C2	Gene expression	resonance	leiomyoma uterine	0.052
2663055	VIM	Gene expression	positive	leiomyoma uterine	0.051

2061492	NR3C2	Binding	imaging	leiomyoma uterine	0.051
10730274	COL4A6	Positive regulation	Mutations	leiomyoma uterine	0.05
15163025	S100B	Gene expression	positive	leiomyoma uterine	0.049
17243163	HMGA2	Binding	one	leiomyoma uterine	0.046
12834940	TNFRSF1A	Positive regulation	increase	leiomyoma uterine	0.046
18538038	MLANA	Positive regulation	requires	leiomyoma uterine	0.046
12637757	PC	Negative regulation	decreases	leiomyoma uterine	0.044
16359541	TP63	Binding	diagnosis	leiomyoma uterine	0.044
10502173	NT5E	Localization	resection	leiomyoma uterine	0.044
10674844	PLP2	Gene expression	resection	leiomyoma uterine	0.044
1535909	FLG2	Localization	located	leiomyoma uterine	0.044
16082144	NR3C2	Regulation	Control	leiomyoma uterine	0.044
14708972	SDHB	Positive regulation	succinate	leiomyoma uterine	0.044
15956493	NR3C2	Gene expression	imaging	leiomyoma uterine	0.043
17197920	JAZF1	Localization	located	leiomyoma uterine	0.043
11960042	GNRHR	Gene expression	received	leiomyoma uterine	0.043
16114791	COL4A6	Protein catabolism	analysis	leiomyoma uterine	0.042
15088185	DAP	Gene expression	product	leiomyoma uterine	0.041
21656660	EZH2	Phosphorylation	phosphorylates	leiomyoma uterine	0.041
12434668	COCH	Gene expression	using	leiomyoma uterine	0.041
2145765	GNRHR	Positive regulation	induce	leiomyoma uterine	0.041
12940465	GNRH1	Regulation	changes	leiomyoma uterine	0.04
11866950	CDKN2A	Positive regulation	Hypermethylation	leiomyoma uterine	0.04
16125217	MCM8	Binding	gained	leiomyoma uterine	0.039
15543383	RAG1	Gene expression	negative	leiomyoma uterine	0.039
17127852	KIT	Gene expression	coexpressed	leiomyoma uterine	0.039
11020519	ESR1	Negative regulation	decreased	leiomyoma uterine	0.039
1709853	GNRH1	Gene expression	synthetic	leiomyoma uterine	0.038
16547985	ELANE	Gene expression	Milwaukee	leiomyoma uterine	0.038
17652533	LMOD1	Gene expression	positive	leiomyoma uterine	0.038
12006687	MRPS18C	Gene expression	imaging	leiomyoma uterine	0.038
16955562	KRT7	Regulation	positivity	leiomyoma uterine	0.036
17403306	TNFRSF1A	Regulation	calculated	leiomyoma uterine	0.034
19083394	TNF	Negative regulation	decreased	leiomyoma uterine	0.034
16676196	ACTC1	Binding	negative	leiomyoma uterine	0.033
19630579	FUS	Localization	release	leiomyoma uterine	0.033

17298155	PGR	Gene expression	levels	leiomyoma uterine	0.033
17346524	CRP	Negative regulation	measurement	leiomyoma uterine	0.033
20969451	RHD	Positive regulation	formulation	leiomyoma uterine	0.033
17222831	IL18	Binding	associated	leiomyoma uterine	0.033
16144493	PLOD1	Localization	secretion	leiomyoma uterine	0.033
11734258	PLOD1	Localization	secretion	leiomyoma uterine	0.033
17350961	MANF	Negative regulation	effect	leiomyoma uterine	0.032
12858116	GNRHR	Negative regulation	blocking	leiomyoma uterine	0.032
15523491	FH	Binding	associated	leiomyoma uterine	0.03
15334541	FH	Negative regulation	predispose	leiomyoma uterine	0.03
19075141	FH	Binding	affected	leiomyoma uterine	0.03
15004438	MMP2	Gene expression	found	leiomyoma uterine	0.028
19063937	PRKAR1A	Positive regulation	mutated	leiomyoma uterine	0.028
17675621	C2	Binding	embolization	leiomyoma uterine	0.027
16358102	VIM	Gene expression	positive	leiomyoma uterine	0.027
16319128	PAPPA	Gene expression	detected	leiomyoma uterine	0.027
17654722	HMGA2	Positive regulation	rearrangements	leiomyoma uterine	0.025
18566572	CLDN1	Regulation	deregulated	leiomyoma uterine	0.024
16686944	RAPH1	Binding	associated	leiomyoma uterine	0.024
12584627	CRP	Positive regulation	increase	leiomyoma uterine	0.018
19135929	SMUG1	Positive regulation	increased	leiomyoma uterine	0.017
16105928	NR3C2	Binding	embolization	leiomyoma uterine	0.01
19075141	FH	Binding	affected	leiomyoma uterine	0.03
15004438	MMP2	Gene expression	found	leiomyoma uterine	0.028
19063937	PRKAR1A	Positive regulation	mutated	leiomyoma uterine	0.028
17675621	C2	Binding	embolization	leiomyoma uterine	0.027
16358102	VIM	Gene expression	positive	leiomyoma uterine	0.027
16319128	PAPPA	Gene expression	detected	leiomyoma uterine	0.027
17654722	HMGA2	Positive regulation	rearrangements	leiomyoma uterine	0.025

Identified known therapeutic gene targets about UFs in Drugbank database

Drugs		Targets		Actions
Drug name	CAS number	Gene name	Uniprot ID	
Oxytocin	50-56-6	OXTR	P30559	Agonist
		OXT	P01178	Binder
Leuprolide	53714-56-0	GNRHR	P30968	Agonist
Terbutaline	23031-25-6	ADRB2	P07550	Agonist
		ADRB3	P13945	Agonist
		ADRB1	P08588	Antagonist
Hydroxyprogestero	630-56-8	PGR	P06401	Agonist
Progesterone	57-83-0	PGR	P06401	Agonist
		ESR1	P03372	Agonist/Inhibitor
		NR3C2	P08235	Antagonist/Agonist
		CYP17A1	P05093	Substrate/Inhibitor
		OPRK1	P41145	Activator/Potentiator
		ORM1	P02763	Binder
		NR3C1	P04150	Partial agonist
		AR	P10275	Agonist/Potentiator
		SHBG	P04278	Binder/Potentiator
		ESR2	Q92731	Agonist/Downregulator
Medroxyprogestero ne acetate	71-58-9	PGR	P06401	Agonist
		ESR1	P03372	Agonist
Misoprostol	59122-46-2	PTGER3	P43115	Agonist
		PTGER2	P43116	Agonist
		PTGER4	P35408	Agonist
Ergotamine	113-15-5	HTR1D	P28221	Agonist
		HTR1B	P28222	Agonist
		HTR2A	P28223	Agonist
		DRD2	P14416	Agonist
		ADRA1A	P35348	Partial agonist
		ADRA1B	P35368	Partial agonist
		ADRA1D	P25100	Partial agonist
		ADRA2A	P08913	Partial agonist
		ADRA2B	P18089	Agonist/Partial agonist
		SLC6A2	P23975	Inhibitor
		DRD1	P21728	agonist
		DRD5	P21918	agonist
		HTR1A	P08908	agonist
		HTR1F	P30939	agonist

		HTR2C	P28335	agonist
		HTR2B	P41595	unknown
Raloxifene	84449-90-1	ESR1	P03372	Agonist
		ESR2	Q92731	Agonist
Larotrectinib	1223403-58-4	NTRK1	P04629	Inhibitor
		NTRK2	Q16620	Inhibitor
		NTRK3	Q16288	Inhibitor
Ivosidenib	1448347-49-6	IDH1	O75874	Inhibitor
D-alpha-Tocopherol acetate	58-95-7	SEC14L4	Q9UDX3	unknown
		SEC14L3	Q9UDX4	unknown
		SEC14L2	O76054	unknown
		NR1I2	O75469	unknown
		PRKCB	P05771	unknown
		ALOX5	P09917	unknown
		PRKCA	P17252	unknown
		DGKA	P23743	unknown
		PPP2CB	P62714	unknown
		PPP2CA	P67775	unknown
Nabumetone	42924-53-8	PTGS2	P35354	Inhibitor
		PTGS1	P23219	Inhibitor
Methylergometrine	113-42-8	DRD1	P21728	Antagonist
		HTR2B	P41595	unknown
Dinoprostone	363-24-6	PTGER2	P43116	Agonist
		PTGER1	P34995	Agonist
		PTGER3	P43115	Agonist
		PTGER4	P35408	Agonist
		PTGDR2	Q9Y5Y4	Agonist
Letrozole	112809-51-5	CYP19A1	P11511	Antagonist
Elagolix	834153-87-6	GNRHR	P30968	unknown
Testosterone cypionate	58-20-8	AR	P10275	Agonist
		ESR1	P03372	unknown
		NR3C2	P08235	unknown
Duvelisib	1201438-56-3	PIK3CG	P48736	Inhibitor
		PIK3CD	O00329	Inhibitor
Mifepristone	84371-65-3	PGR	P06401	Antagonist
		NR3C1	P04150	Antagonist
		KLK3	P07288	unknown
		NR1I2	O75469	unknown
Magnesium sulfate	7487-88-9	CACNG1	Q06432	unknown
		CACNA2D1	P54289	unknown

		CACNA1C	Q13936	unknown
		CACNB1	Q02641	unknown
		CACNB2	Q08289	unknown
		CACNA1S	Q13698	unknown
Salbutamol	18559-94-9	ADRB2	P07550	Agonist
		ADRB1	P08588	Agonist
		ADRB3	P13945	agonist with low
Spironolactone	1952-1-7	NR3C2	P08235	Antagonist
		AR	P10275	Antagonist
		PGR	P06401	Agonist
		NR3C1	P04150	Antagonist
		CYP11B2	P19099	Antagonist
		CYP17A1	P05093	Antagonist
		SRD5A1	P18405	Antagonist
		SRD5A2	P31213	Antagonist
		SRD5A3	Q9H8P0	Antagonist
Sertraline	79617-96-2	SLC6A4	P31645	Inhibitor
		SLC6A3	Q01959	Inhibitor
		PGRMC1	O00264	Antagonist
		SIGMAR1	Q99720	Antagonist
Epinephrine	51-43-4	ADRA1A	P35348	Agonist
		ADRA1B	P35368	Agonist
		ADRB1	P08588	Agonist
		ADRB2	P07550	Agonist
		ADRA2A	P08913	Agonist
		ADRA2B	P18089	Agonist
		ADRA1D	P25100	Antagonist
		TNF	P01375	Antagonist/Agonist
Ibuprofen	15687-27-1	PTGS2	P35354	Inhibitor
		PTGS1	P23219	Inhibitor
		BCL2	P10415	Modulator
		THBD	P07204	Inducer
		FABP2	P12104	Binder
		PPARG	P37231	Activator
		CFTR	P13569	Inhibitor
		PPARA	Q07869	Activator
		GP1BA	P07359	Inducer
		S100A7	P31151	Inducer
Nitroglycerin	55-63-0	NPR1	P16066	Agonist
Ropinirole	91374-21-9	DRD3	P35462	Agonist

		DRD4	P21917	Agonist
		DRD2	P14416	Agonist
		ADRA1A	P35348	Antagonist
		ADRA1B	P35368	Antagonist
		ADRA1D	P25100	Antagonist
		ADRA2A	P08913	Antagonist
		ADRA2B	P18089	Antagonist
		ADRA2C	P18825	Antagonist
Lidocaine	137-58-6	SCN10A	Q9Y5Y9	Inhibitor
		SCN9A	Q15858	Inhibitor
		SCN5A	Q14524	Inhibitor
		EGFR	P00533	Antagonist
		SCN4A	P35499	unknown
		ORM1	P02763	unknown
		ORM2	P19652	unknown
Castor oil	8001-79-4	PTGER3	P43115	Agonist/Activator
		PTGER4	P35408	Agonist
Norethisterone	68-22-4	PGR	P06401	Agonist
Toremifene	89778-26-7	ESR1	P03372	unknown
		SHBG	P04278	unknown
Bazedoxifene	198481-32-2	ESR1	P03372	Antagonist/Agonist
		ESR2	Q92731	unknown
Indapamide	26807-65-8	SLC12A3	P55017	Inhibitor
Dabigatran etexilate	211915-06-9	F2	P00734	Inhibitor
Carboprost	58551-69-2	PTGER1	P34995	Agonist
Goserelin	65807-02-5	LHCGR	P22888	Agonist
		GNRHR	P30968	Agonist
Ulipristal	159811-51-5	PGR	P06401	Modulator
		NR3C1	P04150	Antagonist
		AR	P10275	unknown

Table S4 Detailed information on topological features of XLW-UFs hubs in Cytoscape

SUID	name	selected	shared name	Type
72	IL1R1	FALSE	IL1R1	Target-U
73	GNAS	FALSE	GNAS	Target-U
74	HLA-C	FALSE	HLA-C	Target-U
75	UCHL1	FALSE	UCHL1	Target-U
76	DIH1	FALSE	DIH1	Target-U
77	E2F1	FALSE	E2F1	Target-U
78	ANTXR2	FALSE	ANTXR2	Target-U
79	NCOA1	FALSE	NCOA1	Target-U
80	EZH2	FALSE	EZH2	Target-U
81	CDH1	FALSE	CDH1	Target-U
82	CTNNB1	FALSE	CTNNB1	Target-U
83	TSHR	FALSE	TSHR	Target-U
84	TKT	FALSE	TKT	Target-U
85	AREG	FALSE	AREG	Target-U
86	TMEM37	FALSE	TMEM37	Target-U
87	MIR18A	FALSE	MIR18A	Target-U
88	PAGE4	FALSE	PAGE4	Target-U
89	ANXA1	FALSE	ANXA1	Target-U
90	RPL22P1	FALSE	RPL22P1	Target-U
91	DAP	FALSE	DAP	Target-U
92	CHKB	FALSE	CHKB	Target-U
93	GRK5	FALSE	GRK5	Target-U
94	HSD17B7	FALSE	HSD17B7	Target-U
95	BCR	FALSE	BCR	Target-U
96	TET1	FALSE	TET1	Target-U
97	SELENBP1	FALSE	SELENBP1	Target-U
98	PPP1R15A	FALSE	PPP1R15A	Target-U
99	CNN1	FALSE	CNN1	Target-U
100	EDN2	FALSE	EDN2	Target-U
101	EIF2AK1	FALSE	EIF2AK1	Target-U
102	MED12L	FALSE	MED12L	Target-U
103	GPR182	FALSE	GPR182	Target-U
104	LCK	FALSE	LCK	Target-U
105	FGFR1	FALSE	FGFR1	Target-U
106	OVGP1	FALSE	OVGP1	Target-U
107	IGFBP1	FALSE	IGFBP1	Target-U
108	ACVRL1	FALSE	ACVRL1	Target-U
109	HV1S	FALSE	HV1S	Target-U
110	TCEB1	FALSE	TCEB1	Target-U
111	ADAM17	FALSE	ADAM17	Target-U
112	RHD	FALSE	RHD	Target-U
113	DDT	FALSE	DDT	Target-U
114	LPL	FALSE	LPL	Target-U
115	B3GAT1	FALSE	B3GAT1	Target-U
116	PLOD2	FALSE	PLOD2	Target-U
117	CD99	FALSE	CD99	Target-U
118	FBLN1	FALSE	FBLN1	Target-U
119	TFPI	FALSE	TFPI	Target-U

120	PTN	FALSE	PTN	Target-U
121	RAG1	FALSE	RAG1	Target-U
122	CDK10	FALSE	CDK10	Target-U
123	PCP4	FALSE	PCP4	Target-U
124	CD97	FALSE	CD97	Target-U
125	NCAM1	FALSE	NCAM1	Target-U
126	SLC4A1	FALSE	SLC4A1	Target-U
127	NR4A1	FALSE	NR4A1	Target-U
128	CAPN6	FALSE	CAPN6	Target-U
129	HSD17B2	FALSE	HSD17B2	Target-U
130	EGR1	FALSE	EGR1	Target-U
131	TGFBR2	FALSE	TGFBR2	Target-U
132	TCF4	FALSE	TCF4	Target-U
133	CDK4	FALSE	CDK4	Target-U
134	FZD4	FALSE	FZD4	Target-U
135	MAP3K5	FALSE	MAP3K5	Target-U
136	PLD5	FALSE	PLD5	Target-U
137	BSG	FALSE	BSG	Target-U
138	PDAP1	FALSE	PDAP1	Target-U
139	NT5E	FALSE	NT5E	Target-U
140	PLD2	FALSE	PLD2	Target-U
141	MEN1	FALSE	MEN1	Target-U
142	SIRPA	FALSE	SIRPA	Target-U
143	MRPS18C	FALSE	MRPS18C	Target-U
144	TCEB2	FALSE	TCEB2	Target-U
145	ALK	FALSE	ALK	Target-U
146	CST6	FALSE	CST6	Target-U
147	AP1S2	FALSE	AP1S2	Target-U
148	PLAUR	FALSE	PLAUR	Target-U
149	CYP2C9	FALSE	CYP2C9	Target-U
150	C3	FALSE	C3	Target-U
151	RPL22	FALSE	RPL22	Target-U
152	MLANA	FALSE	MLANA	Target-U
153	BMP6	FALSE	BMP6	Target-U
154	RXRG	FALSE	RXRG	Target-U
155	DNMT1	FALSE	DNMT1	Target-U
156	PLP2	FALSE	PLP2	Target-U
157	TYRO3	FALSE	TYRO3	Target-U
158	LTBP1	FALSE	LTBP1	Target-U
159	MUSK	FALSE	MUSK	Target-U
160	CALCA	FALSE	CALCA	Target-U
161	ZKSCAN7	FALSE	ZKSCAN7	Target-U
162	IL11	FALSE	IL11	Target-U
163	IL17B	FALSE	IL17B	Target-U
164	ANGPT2	FALSE	ANGPT2	Target-U
165	CR1	FALSE	CR1	Target-U
166	GAL	FALSE	GAL	Target-U
167	HSPA5	FALSE	HSPA5	Target-U
168	TP53RK	FALSE	TP53RK	Target-U
169	MRGPRX3	FALSE	MRGPRX3	Target-U
170	HAVCR1	FALSE	HAVCR1	Target-U

171	TERF1	FALSE	TERF1	Target-U
172	CYR61	FALSE	CYR61	Target-U
173	TNFRSF21	FALSE	TNFRSF21	Target-U
174	CCL5	FALSE	CCL5	Target-U
175	AVP	FALSE	AVP	Target-U
176	FZD2	FALSE	FZD2	Target-U
177	SCLC1	FALSE	SCLC1	Target-U
178	PRKAR1A	FALSE	PRKAR1A	Target-U
179	ERBB2	FALSE	ERBB2	Target-U
180	VHL	FALSE	VHL	Target-U
181	CR2	FALSE	CR2	Target-U
182	FBLN5	FALSE	FBLN5	Target-U
183	PTPRU	FALSE	PTPRU	Target-U
184	CXCL14	FALSE	CXCL14	Target-U
185	AKR1C4	FALSE	AKR1C4	Target-U
186	IFNG	FALSE	IFNG	Target-U
187	C2	FALSE	C2	Target-U
188	BNIP3	FALSE	BNIP3	Target-U
189	CSF2	FALSE	CSF2	Target-U
190	PTH	FALSE	PTH	Target-U
191	PPBP	FALSE	PPBP	Target-U
192	MMP11	FALSE	MMP11	Target-U
193	ALDH1A1	FALSE	ALDH1A1	Target-U
194	PECAM1	FALSE	PECAM1	Target-U
195	CP	FALSE	CP	Target-U
196	THBS2	FALSE	THBS2	Target-U
197	TMEFF2	FALSE	TMEFF2	Target-U
198	LGALS3	FALSE	LGALS3	Target-U
199	RGS7	FALSE	RGS7	Target-U
200	IL4	FALSE	IL4	Target-U
201	ADCY10	FALSE	ADCY10	Target-U
202	EREG	FALSE	EREG	Target-U
203	PLA2G2A	FALSE	PLA2G2A	Target-U
204	LDHC	FALSE	LDHC	Target-U
205	PPIG	FALSE	PPIG	Target-U
206	ACOT7	FALSE	ACOT7	Target-U
207	MANF	FALSE	MANF	Target-U
208	WISP2	FALSE	WISP2	Target-U
209	CLDN1	FALSE	CLDN1	Target-U
210	CCL11	FALSE	CCL11	Target-U
211	MYLK	FALSE	MYLK	Target-U
212	SHC1	FALSE	SHC1	Target-U
213	SDHB	FALSE	SDHB	Target-U
214	NFASC	FALSE	NFASC	Target-U
215	CUL2	FALSE	CUL2	Target-U
216	GPER	FALSE	GPER	Target-U
217	ECM2	FALSE	ECM2	Target-U
218	NCOR2	FALSE	NCOR2	Target-U
219	RAD51	FALSE	RAD51	Target-U
220	GNAI1	FALSE	GNAI1	Target-U
221	WT1	FALSE	WT1	Target-U

222	AGER	FALSE	AGER	Target-U
223	PPP4C	FALSE	PPP4C	Target-U
224	IGFBP7	FALSE	IGFBP7	Target-U
225	RORA	FALSE	RORA	Target-U
226	SEMA5A	FALSE	SEMA5A	Target-U
227	KIAA1715	FALSE	KIAA1715	Target-U
228	TEK	FALSE	TEK	Target-U
229	LTBP2	FALSE	LTBP2	Target-U
230	LGALS1	FALSE	LGALS1	Target-U
231	PDCD6	FALSE	PDCD6	Target-U
232	GPLD1	FALSE	GPLD1	Target-U
233	CUX1	FALSE	CUX1	Target-U
234	NBR1	FALSE	NBR1	Target-U
235	TYMP	FALSE	TYMP	Target-U
236	IL1RN	FALSE	IL1RN	Target-U
237	MCM8	FALSE	MCM8	Target-U
238	AKT1	FALSE	AKT1	Target-U
239	TRIB3	FALSE	TRIB3	Target-U
240	LPA	FALSE	LPA	Target-U
241	ADC	FALSE	ADC	Target-U
242	CSF2RB	FALSE	CSF2RB	Target-U
243	GNRHR2	FALSE	GNRHR2	Target-U
244	COCH	FALSE	COCH	Target-U
245	VIPR1	FALSE	VIPR1	Target-U
246	HOXA11	FALSE	HOXA11	Target-U
247	NF2	FALSE	NF2	Target-U
248	AGRP	FALSE	AGRP	Target-U
249	PSMC3IP	FALSE	PSMC3IP	Target-U
250	ZNF382	FALSE	ZNF382	Target-U
251	MYCBP	FALSE	MYCBP	Target-U
252	ITGB1	FALSE	ITGB1	Target-U
253	SERPINA7	FALSE	SERPINA7	Target-U
254	HIST1H3A	FALSE	HIST1H3A	Target-U
255	SLC2A9	FALSE	SLC2A9	Target-U
256	TDGF1	FALSE	TDGF1	Target-U
257	LTA	FALSE	LTA	Target-U
258	PLP1	FALSE	PLP1	Target-U
259	POTED	FALSE	POTED	Target-U
260	TWF1	FALSE	TWF1	Target-U
261	ELANE	FALSE	ELANE	Target-U
262	TPSAB1	FALSE	TPSAB1	Target-U
263	CRABP1	FALSE	CRABP1	Target-U
264	S100A11	FALSE	S100A11	Target-U
265	XRCC3	FALSE	XRCC3	Target-U
266	ND3	FALSE	ND3	Target-U
267	LPAR1	FALSE	LPAR1	Target-U
268	AXL	FALSE	AXL	Target-U
269	OCIAD2	FALSE	OCIAD2	Target-U
270	TMPO	FALSE	TMPO	Target-U
271	IL18	FALSE	IL18	Target-U
272	KRT18	FALSE	KRT18	Target-U

273	CD36	FALSE	CD36	Target-U
274	DNER	FALSE	DNER	Target-U
275	SERPINB1	FALSE	SERPINB1	Target-U
276	SFRP1	FALSE	SFRP1	Target-U
277	EDNRA	FALSE	EDNRA	Target-U
278	GNRH2	FALSE	GNRH2	Target-U
279	AKR1B10	FALSE	AKR1B10	Target-U
280	PML	FALSE	PML	Target-U
281	KRT20	FALSE	KRT20	Target-U
282	FLNB	FALSE	FLNB	Target-U
283	FBN2	FALSE	FBN2	Target-U
284	EGR3	FALSE	EGR3	Target-U
285	GSN	FALSE	GSN	Target-U
286	COX5A	FALSE	COX5A	Target-U
287	SQSTM1	FALSE	SQSTM1	Target-U
288	HK2	FALSE	HK2	Target-U
289	KITLG	FALSE	KITLG	Target-U
290	PIP	FALSE	PIP	Target-U
291	KLF9	FALSE	KLF9	Target-U
292	GAPDH	FALSE	GAPDH	Target-U
293	LPAR3	FALSE	LPAR3	Target-U
294	APOE	FALSE	APOE	Target-U
295	ATP6V0A4	FALSE	ATP6V0A4	Target-U
296	GRPR	FALSE	GRPR	Target-U
297	PLA2G7	FALSE	PLA2G7	Target-U
298	MCL1	FALSE	MCL1	Target-U
299	CCL28	FALSE	CCL28	Target-U
300	SORCS2	FALSE	SORCS2	Target-U
301	CYTB	FALSE	CYTB	Target-U
302	ENPP2	FALSE	ENPP2	Target-U
303	GAS6	FALSE	GAS6	Target-U
304	WNT5B	FALSE	WNT5B	Target-U
305	ETS1	FALSE	ETS1	Target-U
306	XRCC4	FALSE	XRCC4	Target-U
307	PPY	FALSE	PPY	Target-U
308	SNORA62	FALSE	SNORA62	Target-U
309	FLT1	FALSE	FLT1	Target-U
310	EBAG9	FALSE	EBAG9	Target-U
311	COX10	FALSE	COX10	Target-U
312	FBN1	FALSE	FBN1	Target-U
313	DAPK1	FALSE	DAPK1	Target-U
314	VIP	FALSE	VIP	Target-U
315	KRT14	FALSE	KRT14	Target-U
316	LDHA	FALSE	LDHA	Target-U
317	CHRNA5	FALSE	CHRNA5	Target-U
318	ESM1	FALSE	ESM1	Target-U
319	SGK1	FALSE	SGK1	Target-U
320	NCOR1	FALSE	NCOR1	Target-U
321	STS	FALSE	STS	Target-U
322	CYP2B6	FALSE	CYP2B6	Target-U
323	RRS1	FALSE	RRS1	Target-U

324	NPPA	FALSE	NPPA	Target-U
325	PPP2R3A	FALSE	PPP2R3A	Target-U
326	MYOZ2	FALSE	MYOZ2	Target-U
327	LPAR2	FALSE	LPAR2	Target-U
328	MEST	FALSE	MEST	Target-U
329	GRB2	FALSE	GRB2	Target-U
330	IGKV2D-24	FALSE	IGKV2D-24	Target-U
331	FAM215A	FALSE	FAM215A	Target-U
332	CDKN1C	FALSE	CDKN1C	Target-U
333	PLAG1	FALSE	PLAG1	Target-U
334	TNRC6B	FALSE	TNRC6B	Target-U
335	TIPRL	FALSE	TIPRL	Target-U
336	MMP8	FALSE	MMP8	Target-U
337	MIB1	FALSE	MIB1	Target-U
338	DUT	FALSE	DUT	Target-U
339	EDN3	FALSE	EDN3	Target-U
340	HTLVR	FALSE	HTLVR	Target-U
341	COL3A1	FALSE	COL3A1	Target-U
342	BCHE	FALSE	BCHE	Target-U
343	PC	FALSE	PC	Target-U
344	MAPK3	FALSE	MAPK3	Target-U
345	ADIPOQ	FALSE	ADIPOQ	Target-U
346	ATP8A2	FALSE	ATP8A2	Target-U
347	KLF11	FALSE	KLF11	Target-U
348	VIPR2	FALSE	VIPR2	Target-U
349	FLG2	FALSE	FLG2	Target-U
350	LIF	FALSE	LIF	Target-U
351	MUC1	FALSE	MUC1	Target-U
352	BAK1	FALSE	BAK1	Target-U
353	DPT	FALSE	DPT	Target-U
354	KRAS	FALSE	KRAS	Target-U
355	TPO	FALSE	TPO	Target-U
356	ADCYAP1R1	FALSE	ADCYAP1R1	Target-U
357	SLC2A1	FALSE	SLC2A1	Target-U
358	SMUG1	FALSE	SMUG1	Target-U
359	BCL2L1	FALSE	BCL2L1	Target-U
360	CRP	FALSE	CRP	Target-U
361	CTGF	FALSE	CTGF	Target-U
362	RAD51B	FALSE	RAD51B	Target-U
363	HESX1	FALSE	HESX1	Target-U
364	EXOSC10	FALSE	EXOSC10	Target-U
365	PDGFRB	FALSE	PDGFRB	Target-U
366	RBP1	FALSE	RBP1	Target-U
367	GAS1	FALSE	GAS1	Target-U
368	CD68	FALSE	CD68	Target-U
369	TSC2	FALSE	TSC2	Target-U
370	ADM	FALSE	ADM	Target-U
371	GJA1	FALSE	GJA1	Target-U
372	JAZF1	FALSE	JAZF1	Target-U
373	PCYT1A	FALSE	PCYT1A	Target-U
374	HMGA1	FALSE	HMGA1	Target-U

375	HSD17B1	FALSE	HSD17B1	Target-U
376	IGF1R	FALSE	IGF1R	Target-U
377	DDIT3	FALSE	DDIT3	Target-U
378	TNFRSF1A	FALSE	TNFRSF1A	Target-U
379	HMGB1	FALSE	HMGB1	Target-U
380	SUZ12	FALSE	SUZ12	Target-U
381	IL6R	FALSE	IL6R	Target-U
382	DCX	FALSE	DCX	Target-U
383	POLR2E	FALSE	POLR2E	Target-U
384	IL1B	FALSE	IL1B	Target-U
385	KDR	FALSE	KDR	Target-U
386	PIK3R1	FALSE	PIK3R1	Target-U
387	EDNRB	FALSE	EDNRB	Target-U
388	INHA	FALSE	INHA	Target-U
389	CALB2	FALSE	CALB2	Target-U
390	IL6	FALSE	IL6	Target-U
391	CYP2D6	FALSE	CYP2D6	Target-U
392	SPHK1	FALSE	SPHK1	Target-U
393	TIMP1	FALSE	TIMP1	Target-U
394	PTHLH	FALSE	PTHLH	Target-U
395	NES	FALSE	NES	Target-U
396	CCNA2	FALSE	CCNA2	Target-U
397	S100A1	FALSE	S100A1	Target-U
398	CYP26A1	FALSE	CYP26A1	Target-U
399	COL1A1	FALSE	COL1A1	Target-U
400	KRT7	FALSE	KRT7	Target-U
401	FUS	FALSE	FUS	Target-U
402	MAP3K12	FALSE	MAP3K12	Target-U
403	GFAP	FALSE	GFAP	Target-U
404	EPAS1	FALSE	EPAS1	Target-U
405	NCOA3	FALSE	NCOA3	Target-U
406	COL4A2	FALSE	COL4A2	Target-U
407	TIMP2	FALSE	TIMP2	Target-U
408	ACE	FALSE	ACE	Target-U
409	NOS3	FALSE	NOS3	Target-U
410	IRS1	FALSE	IRS1	Target-U
411	PAPPA	FALSE	PAPPA	Target-U
412	PARP1	FALSE	PARP1	Target-U
413	CDKN1A	FALSE	CDKN1A	Target-U
414	MDM2	FALSE	MDM2	Target-U
415	HIF1A	FALSE	HIF1A	Target-U
416	IGF1	FALSE	IGF1	Target-U
417	ENO2	FALSE	ENO2	Target-U
418	THBS1	FALSE	THBS1	Target-U
419	KRT19	FALSE	KRT19	Target-U
420	COL4A6	FALSE	COL4A6	Target-U
421	MMP2	FALSE	MMP2	Target-U
422	NFKB1	FALSE	NFKB1	Target-U
423	MYC	FALSE	MYC	Target-U
424	COL4A5	FALSE	COL4A5	Target-U
425	CTNNBIP1	FALSE	CTNNBIP1	Target-U

426	HRAS	FALSE	HRAS	Target-U
427	PSMD9	FALSE	PSMD9	Target-U
428	CFH	FALSE	CFH	Target-U
429	FOS	FALSE	FOS	Target-U
430	FGF2	FALSE	FGF2	Target-U
431	LMOD1	FALSE	LMOD1	Target-U
432	CRABP2	FALSE	CRABP2	Target-U
433	CDK1	FALSE	CDK1	Target-U
434	XRCC1	FALSE	XRCC1	Target-U
435	LCN2	FALSE	LCN2	Target-U
436	RAPH1	FALSE	RAPH1	Target-U
437	CCL2	FALSE	CCL2	Target-U
438	EDN1	FALSE	EDN1	Target-U
439	MUC16	FALSE	MUC16	Target-U
440	MMP9	FALSE	MMP9	Target-U
441	CGA	FALSE	CGA	Target-U
442	IL8	FALSE	IL8	Target-U
443	MMRN1	FALSE	MMRN1	Target-U
444	ESRRA	FALSE	ESRRA	Target-U
445	HOXA10	FALSE	HOXA10	Target-U
446	SRC	FALSE	SRC	Target-U
447	CDK2	FALSE	CDK2	Target-U
448	CYP1A1	FALSE	CYP1A1	Target-U
449	MMP3	FALSE	MMP3	Target-U
450	COMT	FALSE	COMT	Target-U
451	TP63	FALSE	TP63	Target-U
452	DBT	FALSE	DBT	Target-U
453	PRL	FALSE	PRL	Target-U
454	MMP1	FALSE	MMP1	Target-U
455	CYP1B1	FALSE	CYP1B1	Target-U
456	MAPK1	FALSE	MAPK1	Target-U
457	PDGFRA	FALSE	PDGFRA	Target-U
458	IGF2	FALSE	IGF2	Target-U
459	MED12	FALSE	MED12	Target-U
460	FSHR	FALSE	FSHR	Target-U
461	EDEM1	FALSE	EDEM1	Target-U
462	CCND1	FALSE	CCND1	Target-U
463	TGFB3	FALSE	TGFB3	Target-U
464	None	FALSE	None	Target-U
465	SMN1	FALSE	SMN1	Target-U
466	MKI67	FALSE	MKI67	Target-U
467	MME	FALSE	MME	Target-U
468	EGF	FALSE	EGF	Target-U
469	VEGFA	FALSE	VEGFA	Target-U
470	PCNA	FALSE	PCNA	Target-U
471	CDKN2A	FALSE	CDKN2A	Target-U
472	PLOD1	FALSE	PLOD1	Target-U
473	S100B	FALSE	S100B	Target-U
474	VIM	FALSE	VIM	Target-U
475	ACTC1	FALSE	ACTC1	Target-U
476	HMGA2	FALSE	HMGA2	Target-U

477	GNRH1	FALSE	GNRH1	Target-U
478	TP53	FALSE	TP53	Target-U
479	CD34	FALSE	CD34	Target-U
480	KIT	FALSE	KIT	Target-U
481	DES	FALSE	DES	Target-U
482	FH	FALSE	FH	Target-U
483	LHCGR	FALSE	LHCGR	Target-U
484	F2	FALSE	F2	Target-U
485	SLC12A3	FALSE	SLC12A3	Target-U
486	ORM2	FALSE	ORM2	Target-U
487	SCN4A	FALSE	SCN4A	Target-U
488	EGFR	FALSE	EGFR	Target-U
489	SCN9A	FALSE	SCN9A	Target-U
490	SCN10A	FALSE	SCN10A	Target-U
491	ADRA2C	FALSE	ADRA2C	Target-U
492	DRD4	FALSE	DRD4	Target-U
493	DRD3	FALSE	DRD3	Target-U
494	NPR1	FALSE	NPR1	Target-U
495	S100A7	FALSE	S100A7	Target-U
496	GP1BA	FALSE	GP1BA	Target-U
497	PPARA	FALSE	PPARA	Target-U
498	CFTR	FALSE	CFTR	Target-U
499	PPARG	FALSE	PPARG	Target-U
500	FABP2	FALSE	FABP2	Target-U
501	THBD	FALSE	THBD	Target-U
502	TNF	FALSE	TNF	Target-U
503	SIGMAR1	FALSE	SIGMAR1	Target-U
504	PGRMC1	FALSE	PGRMC1	Target-U
505	SLC6A3	FALSE	SLC6A3	Target-U
506	SRD5A3	FALSE	SRD5A3	Target-U
507	SRD5A2	FALSE	SRD5A2	Target-U
508	SRD5A1	FALSE	SRD5A1	Target-U
509	CYP11B2	FALSE	CYP11B2	Target-U
510	KLK3	FALSE	KLK3	Target-U
511	PIK3CD	FALSE	PIK3CD	Target-U
512	CYP19A1	FALSE	CYP19A1	Target-U
513	PTGDR2	FALSE	PTGDR2	Target-U
514	PTGER1	FALSE	PTGER1	Target-U
515	PPP2CA	FALSE	PPP2CA	Target-U
516	PPP2CB	FALSE	PPP2CB	Target-U
517	DGKA	FALSE	DGKA	Target-U
518	ALOX5	FALSE	ALOX5	Target-U
519	PRKCB	FALSE	PRKCB	Target-U
520	NR1I2	FALSE	NR1I2	Target-U
521	SEC14L2	FALSE	SEC14L2	Target-U
522	SEC14L3	FALSE	SEC14L3	Target-U
523	SEC14L4	FALSE	SEC14L4	Target-U
524	IDH1	FALSE	IDH1	Target-U
525	NTRK3	FALSE	NTRK3	Target-U
526	NTRK2	FALSE	NTRK2	Target-U
527	NTRK1	FALSE	NTRK1	Target-U

528	HTR2B	FALSE	HTR2B	Target-U
529	HTR2C	FALSE	HTR2C	Target-U
530	HTR1F	FALSE	HTR1F	Target-U
531	DRD5	FALSE	DRD5	Target-U
532	SLC6A2	FALSE	SLC6A2	Target-U
533	ADRA2B	FALSE	ADRA2B	Target-U
534	ADRA2A	FALSE	ADRA2A	Target-U
535	HTR1B	FALSE	HTR1B	Target-U
536	HTR1D	FALSE	HTR1D	Target-U
537	PTGER4	FALSE	PTGER4	Target-U
538	PTGER2	FALSE	PTGER2	Target-U
539	PTGER3	FALSE	PTGER3	Target-U
540	ESR2	FALSE	ESR2	Target-U
541	SHBG	FALSE	SHBG	Target-U
542	AR	FALSE	AR	Target-U
543	NR3C1	FALSE	NR3C1	Target-U
544	ORM1	FALSE	ORM1	Target-U
545	OPRK1	FALSE	OPRK1	Target-U
546	CYP17A1	FALSE	CYP17A1	Target-U
547	ESR1	FALSE	ESR1	Target-U
548	ADRB1	FALSE	ADRB1	Target-U
549	ADRB3	FALSE	ADRB3	Target-U
550	GNRHR	FALSE	GNRHR	Target-U
551	OXT	FALSE	OXT	Target-U
552	OXTR	FALSE	OXTR	Target-U
553	Uterine Fibroid	FALSE	Uterine Fibroid	Disease
554	FDPS	FALSE	FDPS	Target-X
555	HTR1A	FALSE	HTR1A	Target-X+U
556	SLC18A2	FALSE	SLC18A2	Target-X
557	SLC12A4	FALSE	SLC12A4	Target-X
558	SLC12A1	FALSE	SLC12A1	Target-X
559	SLC12A6	FALSE	SLC12A6	Target-X
560	SLC12A5	FALSE	SLC12A5	Target-X
561	SLC12A7	FALSE	SLC12A7	Target-X
562	SLC12A2	FALSE	SLC12A2	Target-X
563	CHRNB4	FALSE	CHRNB4	Target-X
564	CHRNB2	FALSE	CHRNB2	Target-X
565	CHRNA3	FALSE	CHRNA3	Target-X
566	P2RX3	FALSE	P2RX3	Target-X
567	P2RX2	FALSE	P2RX2	Target-X
568	KCNMA1	FALSE	KCNMA1	Target-X
569	DMTN	FALSE	DMTN	Target-X
570	FOXL2	FALSE	FOXL2	Target-X
571	CACNG1	FALSE	CACNG1	Target-X+U
572	CACNB2	FALSE	CACNB2	Target-X+U
573	CACNA1C	FALSE	CACNA1C	Target-X+U
574	CACNA1S	FALSE	CACNA1S	Target-X+U
575	CACNA2D1	FALSE	CACNA2D1	Target-X+U
576	CACNB1	FALSE	CACNB1	Target-X+U
577	ATP1A1	FALSE	ATP1A1	Target-X
578	TF	FALSE	TF	Target-X

579	NR3C2	FALSE	NR3C2	Target-X+U
580	DRD2	FALSE	DRD2	Target-X+U
581	ADRA1D	FALSE	ADRA1D	Target-X+U
582	ACHE	FALSE	ACHE	Target-X
583	OPRD1	FALSE	OPRD1	Target-X
584	CA2	FALSE	CA2	Target-X
585	CHRM5	FALSE	CHRM5	Target-X
586	RXRA	FALSE	RXRA	Target-X+U
587	MAP2	FALSE	MAP2	Target-X
588	PON1	FALSE	PON1	Target-X+U
589	TGFB1	FALSE	TGFB1	Target-X+U
590	PRKCA	FALSE	PRKCA	Target-X+U
591	CASP8	FALSE	CASP8	Target-X
592	CASP3	FALSE	CASP3	Target-X+U
593	JUN	FALSE	JUN	Target-X+U
594	CASP9	FALSE	CASP9	Target-X+U
595	BAX	FALSE	BAX	Target-X+U
596	BCL2	FALSE	BCL2	Target-X+U
597	CYP101A1	FALSE	CYP101A1	Target-X
598	CHRNA7	FALSE	CHRNA7	Target-X
599	GABRA1	FALSE	GABRA1	Target-X
600	OPRM1	FALSE	OPRM1	Target-X
601	SLC6A4	FALSE	SLC6A4	Target-X+U
602	CHRNA2	FALSE	CHRNA2	Target-X
603	ADRB2	FALSE	ADRB2	Target-X+U
604	ADRA1B	FALSE	ADRA1B	Target-X+U
605	CHRM2	FALSE	CHRM2	Target-X
606	GABRA3	FALSE	GABRA3	Target-X
607	ADRA1A	FALSE	ADRA1A	Target-X+U
608	GABRA5	FALSE	GABRA5	Target-X
609	HTR2A	FALSE	HTR2A	Target-X+U
610	PDE3A	FALSE	PDE3A	Target-X
611	CHRM4	FALSE	CHRM4	Target-X
612	GABRA2	FALSE	GABRA2	Target-X
613	SCN5A	FALSE	SCN5A	Target-X+U
614	CHRM1	FALSE	CHRM1	Target-X
615	CHRM3	FALSE	CHRM3	Target-X
616	DRD1	FALSE	DRD1	Target-X+U
617	PRKACA	FALSE	PRKACA	Target-X
618	KCNH2	FALSE	KCNH2	Target-X
619	PIK3CG	FALSE	PIK3CG	Target-X+U
620	HSP90AB1	FALSE	HSP90AB1	Target-X
621	PTGS2	FALSE	PTGS2	Target-X+U
622	PTGS1	FALSE	PTGS1	Target-X+U
623	NCOA2	FALSE	NCOA2	Target-X+U
624	PGR	FALSE	PGR	Target-X+U
625	Peiminoside_qt	FALSE	Peiminoside_qt	Compound
626	Peiminoside	FALSE	Peiminoside	Compound
627	Peiminine	FALSE	Peiminine	Compound
628	Chaksine	FALSE	Chaksine	Compound
629	OSI-2040	FALSE	OSI-2040	Compound

630	Solatubin	FALSE	Solatubin	Compound
631	6-Methoxyl-2-acetyl-3-methyl-1,4-naphthoquinone-8-O-beta-D-glucopyranoside_qt	FALSE	6-Methoxyl-2-acetyl-3-methyl-1,4-naphthoquinone-8-O-beta-D-glucopyranoside_qt	Compound
632	6-Methoxyl-2-acetyl-3-methyl-1,4-naphthoquinone-8-O-beta-D-glucopyranoside	FALSE	6-Methoxyl-2-acetyl-3-methyl-1,4-naphthoquinone-8-O-beta-D-glucopyranoside	Compound
633	Verticine	FALSE	Verticine	Compound
634	Ziebeimine	FALSE	Ziebeimine	Compound
635	Zhebeiresinol	FALSE	Zhebeiresinol	Compound-E
636	Pelargonidin-3,5-diglucoside	FALSE	Pelargonidin-3,5-diglucoside	Compound
637	Peimisine	FALSE	Peimisine	Compound
638	syringaresinol	FALSE	syringaresinol	Compound
639	2,5-Dimethoxybenzoquinone	FALSE	2,5-Dimethoxybenzoquinone	Compound
640	pelargonidin	FALSE	pelargonidin	Compound
641	Zhebeimu	FALSE	Zhebeimu	Herb
642	Cedrol	FALSE	Cedrol	Compound
643	cistanoside D	FALSE	cistanoside D	Compound
644	7-hydroxy-9-hydroxymethyl-3-oxo-	FALSE	7-hydroxy-9-hydroxymethyl-3-oxo-	Compound
645	harpagide	FALSE	harpagide	Compound
646	6-O-methylcatalpol_qt	FALSE	6-O-methylcatalpol_qt	Compound
647	6-O-methylcatalpol	FALSE	6-O-methylcatalpol	Compound
648	6'-O-cinnamoylharpagirle_qt 2	FALSE	6'-O-cinnamoylharpagirle_qt 2	Compound
649	6'-O-cinnamoylharpagirle	FALSE	6'-O-cinnamoylharpagirle	Compound
650	5-(methylthio)valeronitrile	FALSE	5-(methylthio)valeronitrile	Compound
651	geniposide_qt	FALSE	geniposide_qt	Compound
652	geniposide	FALSE	geniposide	Compound
653	catapol	FALSE	catapol	Compound
654	harpagoside_qt	FALSE	harpagoside_qt	Compound
655	harpagoside	FALSE	harpagoside	Compound
656	scropolioside D_qt	FALSE	scropolioside D_qt	Compound
657	scropolioside D	FALSE	scropolioside D	Compound
658	14-deoxy-12(R)-sulfoandrographolide	FALSE	14-deoxy-12(R)-sulfoandrographolide	Compound
659	scropolioside A_qt	FALSE	scropolioside A_qt	Compound

660	scropolioside A	FALSE	scropolioside A	Compound
661	angroside C	FALSE	angroside C	Compound
662	HMF	FALSE	HMF	Compound
663	Homovanillyl alcohol	FALSE	Homovanillyl alcohol	Compound
664	paeonioflorin	FALSE	paeonioflorin	Compound
665	oleic acid	FALSE	oleic acid	Compound
666	aucubigenin	FALSE	aucubigenin	Compound
667	ursolic acid	FALSE	ursolic acid	Compound
668	Crystal VI	FALSE	Crystal VI	Compound
669	FERULIC ACID (CIS)	FALSE	FERULIC ACID (CIS)	Compound
670	aucubin	FALSE	aucubin	Compound
671	FER	FALSE	FER	Compound
672	sitosterol	FALSE	sitosterol	Compound-E
673	beta-sitosterol	FALSE	beta-sitosterol	Compound-E
674	Sitogluside	FALSE	Sitogluside	Compound
675	lupeol	FALSE	lupeol	Compound
676	succinic acid	FALSE	succinic acid	Compound
677	5-(2-hydroxyethyl)-2-methoxyphenol	FALSE	5-(2-hydroxyethyl)-2-methoxyphenol	Compound
678	catapol_qt	FALSE	catapol_qt	Compound
679	cinnamic acid	FALSE	cinnamic acid	Compound
680	caffeic acid	FALSE	caffeic acid	Compound
681	sugiol	FALSE	sugiol	Compound-E
682	paeoniflorin_qt	FALSE	paeoniflorin_qt	Compound
683	p-MCA	FALSE	p-MCA	Compound
684	decaffeoylacteoside	FALSE	decaffeoylacteoside	Compound
685	acteoside	FALSE	acteoside	Compound
686	hydroxytyrosol	FALSE	hydroxytyrosol	Compound
687	cis-Zimtsaeure	FALSE	cis-Zimtsaeure	Compound
688	vanillic acid	FALSE	vanillic acid	Compound
689	Xuanshen	FALSE	Xuanshen	Herb
690	Calcium Phosphate	FALSE	Calcium Phosphate	Compound-E
691	Calcium Carbonate	FALSE	Calcium Carbonate	Compound-E
692	Silicon	FALSE	Silicon	Compound-E
693	Calcium Sulphate	FALSE	Calcium Sulphate	Compound-E
694	Aluminum	FALSE	Aluminum	Compound-E
695	Shengmuli	FALSE	Shengmuli	Herb

Table S5 GO enrichment and KEGG pathway analysis about target genes in XLW-treated UFs

Enrichment term	Term ID	Observed gene count	Background gene count	FDR
GO cellular composition	GO:0005901	5	74	9.03E-06
GO cellular composition	GO:0044459	15	2651	9.03E-06
GO cellular composition	GO:0045121	7	300	9.03E-06
GO cellular composition	GO:0030315	4	45	1.16E-05
GO cellular composition	GO:0005887	11	1564	3.07E-05
GO cellular composition	GO:0098805	11	1554	3.07E-05
GO cellular composition	GO:0031674	4	136	0.00045
GO cellular composition	GO:0098590	8	1061	0.00053
GO cellular composition	GO:1990454	2	6	0.0006
GO cellular composition	GO:0005886	16	5159	0.0011
GO cellular composition	GO:0044425	18	6517	0.0011
GO cellular composition	GO:0016020	20	8420	0.0017
GO cellular composition	GO:0031090	12	3337	0.0029
GO cellular composition	GO:0098797	5	502	0.0032
GO cellular composition	GO:0030018	3	122	0.0041
GO cellular composition	GO:0043229	23	12193	0.0069
GO cellular composition	GO:0012505	13	4347	0.0077
GO cellular composition	GO:0098796	6	1047	0.0112
GO cellular composition	GO:0016328	2	55	0.0144
GO cellular composition	GO:0034703	3	206	0.0144
GO cellular composition	GO:0032991	13	4792	0.0159
GO cellular composition	GO:0044422	19	9111	0.0159
GO cellular composition	GO:0005622	24	14286	0.0212
GO cellular composition	GO:0005737	21	11238	0.0247
GO cellular composition	GO:0043227	21	11244	0.0247
GO cellular composition	GO:0016021	13	5155	0.0264
GO cellular composition	GO:0060170	2	85	0.0264
GO cellular composition	GO:0005783	7	1796	0.0277
GO cellular composition	GO:0043235	3	305	0.03
GO cellular composition	GO:0005789	5	1022	0.0354
GO cellular composition	GO:0097458	6	1449	0.0354
GO cellular composition	GO:0098827	5	1025	0.0354
GO cellular composition	GO:0044464	25	16244	0.0363
GO cellular composition	GO:0097730	2	128	0.0432
GO cellular composition	GO:0044444	18	9377	0.0464
GO cellular composition	GO:0031967	5	1146	0.0474
GO cellular composition	GO:0043005	5	1142	0.0474
GO molecular function	GO:0008227	6	48	1.59E-08
GO molecular function	GO:0004935	4	10	2.90E-07
GO molecular function	GO:0070405	5	66	2.57E-06
GO molecular function	GO:0004937	3	3	2.59E-06
GO molecular function	GO:0046983	11	1301	1.02E-05
GO molecular function	GO:0038023	11	1429	2.24E-05
GO molecular function	GO:0005515	20	6605	9.07E-05
GO molecular function	GO:1901338	3	20	9.07E-05
GO molecular function	GO:0004930	8	824	0.00015
GO molecular function	GO:0004666	2	2	0.00022

GO molecular function	GO:0030594	4	107	0.00025
GO molecular function	GO:0046982	6	519	0.00083
GO molecular function	GO:0003707	3	59	0.0012
GO molecular function	GO:0042803	7	830	0.0012
GO molecular function	GO:0097200	2	10	0.0017
GO molecular function	GO:0004952	2	12	0.0018
GO molecular function	GO:0008331	2	11	0.0018
GO molecular function	GO:0051378	2	11	0.0018
GO molecular function	GO:0035240	2	13	0.002
GO molecular function	GO:0043169	14	4170	0.002
GO molecular function	GO:0050998	2	13	0.002
GO molecular function	GO:0019899	10	2197	0.0022
GO molecular function	GO:0042802	9	1754	0.0022
GO molecular function	GO:0004993	2	22	0.0041
GO molecular function	GO:0005102	8	1513	0.0041
GO molecular function	GO:0005488	23	11878	0.0048
GO molecular function	GO:0004601	2	40	0.0109
GO molecular function	GO:0008289	5	673	0.0115
GO molecular function	GO:0019904	5	706	0.0132
GO molecular function	GO:0004879	2	50	0.0138
GO molecular function	GO:0005244	3	198	0.0138
GO molecular function	GO:0005516	3	194	0.0138
GO molecular function	GO:0015459	2	51	0.0138
GO molecular function	GO:0046873	4	458	0.016
GO molecular function	GO:0015267	4	461	0.0161
GO molecular function	GO:0005496	2	88	0.0324
GO molecular function	GO:0051213	2	88	0.0324
GO molecular function	GO:0044877	5	968	0.0363
GO molecular function	GO:0005261	3	316	0.0383
GO molecular function	GO:0097159	13	5382	0.0407
GO molecular function	GO:0016922	2	111	0.0418
GO molecular function	GO:0022857	5	1049	0.0429
GO biological process	GO:0008015	14	373	9.39E-15
GO biological process	GO:0003018	10	162	4.78E-12
GO biological process	GO:0097746	9	129	3.58E-11
GO biological process	GO:0097756	7	39	4.00E-11
GO biological process	GO:0043269	12	618	1.02E-09
GO biological process	GO:0042310	6	31	1.20E-09
GO biological process	GO:0014070	13	873	2.04E-09
GO biological process	GO:0003008	16	1827	6.22E-09
GO biological process	GO:0010033	18	2815	2.28E-08
GO biological process	GO:0071407	10	505	5.24E-08
GO biological process	GO:0090257	8	224	5.24E-08
GO biological process	GO:0010959	9	360	5.98E-08
GO biological process	GO:0044057	10	516	5.98E-08
GO biological process	GO:0051716	23	6212	6.77E-08
GO biological process	GO:0042221	20	4153	7.60E-08
GO biological process	GO:1903522	8	249	8.25E-08
GO biological process	GO:0007204	8	251	8.36E-08
GO biological process	GO:0006874	9	388	8.64E-08
GO biological process	GO:0006937	7	156	1.05E-07

GO biological process	GO:0045907	5	33	1.06E-07
GO biological process	GO:0001993	4	9	1.27E-07
GO biological process	GO:0009719	13	1353	1.66E-07
GO biological process	GO:0008217	7	177	1.78E-07
GO biological process	GO:0023052	21	5108	1.78E-07
GO biological process	GO:0007188	7	183	2.09E-07
GO biological process	GO:0071495	12	1106	2.09E-07
GO biological process	GO:0051049	14	1732	2.21E-07
GO biological process	GO:0007154	21	5219	2.33E-07
GO biological process	GO:0050896	24	7824	2.72E-07
GO biological process	GO:0042493	11	900	3.06E-07
GO biological process	GO:0048545	8	324	3.10E-07
GO biological process	GO:0007200	6	109	3.33E-07
GO biological process	GO:0065008	18	3559	3.53E-07
GO biological process	GO:0051282	6	113	3.73E-07
GO biological process	GO:0050801	10	708	4.17E-07
GO biological process	GO:0035556	13	1528	4.45E-07
GO biological process	GO:0032225	4	17	5.96E-07
GO biological process	GO:0071880	4	17	5.96E-07
GO biological process	GO:0042127	13	1594	6.81E-07
GO biological process	GO:0006940	5	60	7.81E-07
GO biological process	GO:0006936	7	244	8.66E-07
GO biological process	GO:0050805	5	62	8.66E-07
GO biological process	GO:0032501	22	6507	9.18E-07
GO biological process	GO:0007267	11	1073	1.19E-06
GO biological process	GO:0042391	8	408	1.19E-06
GO biological process	GO:0009410	7	262	1.26E-06
GO biological process	GO:0033993	10	825	1.28E-06
GO biological process	GO:0009725	10	854	1.71E-06
GO biological process	GO:0034765	8	434	1.74E-06
GO biological process	GO:1901700	12	1427	1.75E-06
GO biological process	GO:0001994	3	4	2.15E-06
GO biological process	GO:0010604	16	3081	2.33E-06
GO biological process	GO:1904062	7	294	2.33E-06
GO biological process	GO:0007165	19	4738	2.40E-06
GO biological process	GO:0042592	12	1491	2.50E-06
GO biological process	GO:0032270	12	1496	2.56E-06
GO biological process	GO:0009636	8	468	2.60E-06
GO biological process	GO:0070887	15	2672	2.75E-06
GO biological process	GO:0048518	20	5459	2.80E-06
GO biological process	GO:0007189	5	88	3.10E-06
GO biological process	GO:0050804	7	316	3.32E-06
GO biological process	GO:0001659	4	33	3.69E-06
GO biological process	GO:0003073	5	92	3.69E-06
GO biological process	GO:0045987	4	33	3.69E-06
GO biological process	GO:0048522	19	4898	3.69E-06
GO biological process	GO:0006811	11	1292	5.22E-06
GO biological process	GO:0007610	8	541	6.43E-06
GO biological process	GO:0043279	5	106	6.43E-06
GO biological process	GO:0007613	5	109	7.17E-06
GO biological process	GO:0009628	10	1052	7.81E-06

GO biological process	GO:0001932	11	1370	8.64E-06
GO biological process	GO:0072359	9	807	9.25E-06
GO biological process	GO:0051924	6	236	1.09E-05
GO biological process	GO:0007611	6	237	1.10E-05
GO biological process	GO:0032355	5	126	1.29E-05
GO biological process	GO:0050806	5	126	1.29E-05
GO biological process	GO:0006939	4	51	1.44E-05
GO biological process	GO:0043270	6	252	1.50E-05
GO biological process	GO:0071310	13	2219	1.51E-05
GO biological process	GO:0010243	9	876	1.65E-05
GO biological process	GO:0009314	7	425	1.68E-05
GO biological process	GO:0009605	12	1857	1.77E-05
GO biological process	GO:0051050	9	892	1.87E-05
GO biological process	GO:0019932	6	266	1.92E-05
GO biological process	GO:1903169	5	146	2.34E-05
GO biological process	GO:0051966	4	61	2.58E-05
GO biological process	GO:0051239	14	2788	2.62E-05
GO biological process	GO:0009416	6	290	3.01E-05
GO biological process	GO:0060402	4	65	3.20E-05
GO biological process	GO:0009653	12	1992	3.48E-05
GO biological process	GO:0071417	7	485	3.66E-05
GO biological process	GO:0032268	13	2486	4.84E-05
GO biological process	GO:0051173	14	2946	4.84E-05
GO biological process	GO:0055117	4	74	4.92E-05
GO biological process	GO:0043410	7	512	4.94E-05
GO biological process	GO:0065007	25	11740	5.05E-05
GO biological process	GO:0051279	4	75	5.06E-05
GO biological process	GO:0048148	3	20	5.53E-05
GO biological process	GO:0001963	3	21	6.22E-05
GO biological process	GO:0002028	4	82	6.86E-05
GO biological process	GO:0031325	14	3060	7.07E-05
GO biological process	GO:0070374	5	196	8.06E-05
GO biological process	GO:0071383	5	197	8.20E-05
GO biological process	GO:0032502	18	5401	8.35E-05
GO biological process	GO:0008016	5	201	8.83E-05
GO biological process	GO:0051588	4	92	9.99E-05
GO biological process	GO:0097191	4	93	0.0001
GO biological process	GO:0002027	4	95	0.00011
GO biological process	GO:0044706	5	211	0.00011
GO biological process	GO:0006812	8	866	0.00013
GO biological process	GO:0008284	8	878	0.00014
GO biological process	GO:0021761	4	101	0.00014
GO biological process	GO:0001508	4	104	0.00015
GO biological process	GO:0051240	10	1551	0.00016
GO biological process	GO:0097192	3	31	0.00016
GO biological process	GO:0021537	5	237	0.00018
GO biological process	GO:0006816	5	242	0.00019
GO biological process	GO:0043029	3	33	0.00019
GO biological process	GO:0001934	8	941	0.00021
GO biological process	GO:0010648	9	1255	0.00021
GO biological process	GO:0023057	9	1258	0.00021

GO biological process	GO:0030001	7	664	0.00021
GO biological process	GO:0050789	24	11116	0.00021
GO biological process	GO:0008285	7	669	0.00022
GO biological process	GO:0032227	2	3	0.00022
GO biological process	GO:0098912	2	3	0.00022
GO biological process	GO:1903530	7	672	0.00022
GO biological process	GO:0007568	5	255	0.00023
GO biological process	GO:0010647	10	1631	0.00023
GO biological process	GO:0023056	10	1638	0.00023
GO biological process	GO:0048513	13	2926	0.00023
GO biological process	GO:1902533	8	959	0.00023
GO biological process	GO:0060359	4	122	0.00024
GO biological process	GO:0060322	7	692	0.00026
GO biological process	GO:0086010	3	42	0.00032
GO biological process	GO:0007275	16	4726	0.00034
GO biological process	GO:0015850	4	134	0.00034
GO biological process	GO:0048871	5	283	0.00034
GO biological process	GO:0071214	5	282	0.00034
GO biological process	GO:0007507	6	485	0.00035
GO biological process	GO:0071396	6	486	0.00035
GO biological process	GO:0001666	5	288	0.00036
GO biological process	GO:0010035	6	491	0.00036
GO biological process	GO:0051384	4	138	0.00036
GO biological process	GO:0009411	4	139	0.00037
GO biological process	GO:0045776	3	46	0.00038
GO biological process	GO:0086045	2	5	0.00038
GO biological process	GO:0001505	5	295	0.00039
GO biological process	GO:0043271	4	143	0.00039
GO biological process	GO:0097190	5	295	0.00039
GO biological process	GO:0048609	7	766	0.00042
GO biological process	GO:0098900	3	48	0.00042
GO biological process	GO:0043525	3	49	0.00044
GO biological process	GO:0008219	8	1080	0.00046
GO biological process	GO:0035690	5	310	0.00047
GO biological process	GO:0021853	2	6	0.00048
GO biological process	GO:0034349	2	6	0.00048
GO biological process	GO:0050678	5	311	0.00048
GO biological process	GO:0071478	4	153	0.00048
GO biological process	GO:0071466	4	157	0.00052
GO biological process	GO:0001101	5	323	0.00054
GO biological process	GO:0006367	4	162	0.00057
GO biological process	GO:0043536	3	55	0.00057
GO biological process	GO:0008635	2	7	0.00058
GO biological process	GO:0014062	2	7	0.00058
GO biological process	GO:0051726	8	1129	0.00058
GO biological process	GO:0048468	9	1493	0.0006
GO biological process	GO:0010038	5	339	0.00065
GO biological process	GO:0010646	13	3327	0.00068
GO biological process	GO:0051584	2	8	0.00069
GO biological process	GO:0060135	3	60	0.00069
GO biological process	GO:0023051	13	3360	0.00074

GO biological process	GO:0007417	7	861	0.00076
GO biological process	GO:0032870	6	585	0.00078
GO biological process	GO:0097164	4	179	0.00078
GO biological process	GO:0007565	4	180	0.00079
GO biological process	GO:0045136	2	9	0.00082
GO biological process	GO:0051926	3	65	0.00082
GO biological process	GO:0019222	18	6516	0.00083
GO biological process	GO:0051171	17	5827	0.00084
GO biological process	GO:0051270	7	886	0.00086
GO biological process	GO:0070588	4	189	0.0009
GO biological process	GO:0006970	3	68	0.00091
GO biological process	GO:0019216	5	373	0.00092
GO biological process	GO:0030154	13	3457	0.00093
GO biological process	GO:0030432	2	10	0.00093
GO biological process	GO:0032025	2	10	0.00093
GO biological process	GO:0045787	5	376	0.00093
GO biological process	GO:0055085	8	1235	0.00094
GO biological process	GO:0051649	9	1616	0.00096
GO biological process	GO:0098662	6	618	0.00096
GO biological process	GO:0007186	8	1247	0.00099
GO biological process	GO:0006915	7	915	0.001
GO biological process	GO:0019371	2	11	0.0011
GO biological process	GO:0034103	3	73	0.0011
GO biological process	GO:0044703	7	923	0.0011
GO biological process	GO:0045760	2	11	0.0011
GO biological process	GO:0050877	8	1271	0.0011
GO biological process	GO:0080090	17	5982	0.0011
GO biological process	GO:0007420	6	650	0.0012
GO biological process	GO:0009612	4	210	0.0012
GO biological process	GO:0015872	2	12	0.0012
GO biological process	GO:0018958	3	78	0.0012
GO biological process	GO:0034644	3	78	0.0012
GO biological process	GO:0043281	4	209	0.0012
GO biological process	GO:0048731	14	4144	0.0012
GO biological process	GO:0031323	17	6082	0.0013
GO biological process	GO:0043502	3	81	0.0013
GO biological process	GO:0060158	2	13	0.0013
GO biological process	GO:0060255	17	6072	0.0013
GO biological process	GO:2000177	3	79	0.0013
GO biological process	GO:0008283	6	676	0.0014
GO biological process	GO:0044093	9	1713	0.0014
GO biological process	GO:0051952	3	82	0.0014
GO biological process	GO:1901615	5	420	0.0014
GO biological process	GO:2001023	3	83	0.0014
GO biological process	GO:0001976	2	14	0.0015
GO biological process	GO:0006919	3	85	0.0015
GO biological process	GO:0007625	2	14	0.0015
GO biological process	GO:0014854	2	14	0.0015
GO biological process	GO:0019372	2	14	0.0015
GO biological process	GO:0034220	7	995	0.0015
GO biological process	GO:0045986	2	14	0.0015

GO biological process	GO:0051967	2	14	0.0015
GO biological process	GO:0060134	2	14	0.0015
GO biological process	GO:0002029	2	15	0.0016
GO biological process	GO:1902531	9	1764	0.0016
GO biological process	GO:0043085	8	1381	0.0017
GO biological process	GO:0043266	3	92	0.0017
GO biological process	GO:0051704	10	2222	0.0017
GO biological process	GO:0031667	5	455	0.0018
GO biological process	GO:0048771	3	94	0.0018
GO biological process	GO:0021756	2	17	0.0019
GO biological process	GO:0050790	10	2249	0.0019
GO biological process	GO:0006950	12	3267	0.002
GO biological process	GO:0048511	4	250	0.002
GO biological process	GO:0003085	2	18	0.0021
GO biological process	GO:0006690	3	99	0.0021
GO biological process	GO:0031329	6	743	0.0021
GO biological process	GO:0050794	22	10484	0.0021
GO biological process	GO:0030220	2	19	0.0022
GO biological process	GO:2000178	2	19	0.0022
GO biological process	GO:0019953	6	764	0.0023
GO biological process	GO:0046883	4	261	0.0023
GO biological process	GO:0060341	6	766	0.0023
GO biological process	GO:0042053	2	20	0.0024
GO biological process	GO:0035637	3	110	0.0026
GO biological process	GO:0060444	2	21	0.0026
GO biological process	GO:0045859	6	788	0.0027
GO biological process	GO:0030728	2	22	0.0028
GO biological process	GO:0051968	2	22	0.0028
GO biological process	GO:0098664	2	22	0.0028
GO biological process	GO:0045860	5	517	0.003
GO biological process	GO:0001764	3	118	0.0031
GO biological process	GO:0001782	2	24	0.0033
GO biological process	GO:0043010	4	292	0.0033
GO biological process	GO:0045598	3	120	0.0033
GO biological process	GO:0007190	2	25	0.0035
GO biological process	GO:0032496	4	298	0.0035
GO biological process	GO:0003231	3	125	0.0036
GO biological process	GO:0007628	2	26	0.0037
GO biological process	GO:0009611	5	547	0.0037
GO biological process	GO:0055017	2	26	0.0037
GO biological process	GO:0010575	2	27	0.0039
GO biological process	GO:0042417	2	27	0.0039
GO biological process	GO:1902692	2	27	0.0039
GO biological process	GO:0006810	13	4130	0.004
GO biological process	GO:0009887	6	865	0.004
GO biological process	GO:0014059	2	28	0.004
GO biological process	GO:0015718	3	131	0.004
GO biological process	GO:0016572	2	28	0.004
GO biological process	GO:0043401	3	131	0.004
GO biological process	GO:0048584	9	2054	0.004
GO biological process	GO:0050873	2	28	0.004

GO biological process	GO:0071549	2	28	0.004
GO biological process	GO:1904064	3	131	0.004
GO biological process	GO:0006693	2	29	0.0042
GO biological process	GO:0007612	3	137	0.0042
GO biological process	GO:0007623	3	137	0.0042
GO biological process	GO:0009987	25	14652	0.0042
GO biological process	GO:0010941	8	1638	0.0042
GO biological process	GO:0015721	2	29	0.0042
GO biological process	GO:0033135	3	136	0.0042
GO biological process	GO:0045834	3	135	0.0042
GO biological process	GO:0051336	7	1238	0.0042
GO biological process	GO:0071470	2	29	0.0042
GO biological process	GO:0001662	2	30	0.0043
GO biological process	GO:0007202	2	30	0.0043
GO biological process	GO:0009746	3	138	0.0043
GO biological process	GO:0010613	2	30	0.0043
GO biological process	GO:0033280	2	30	0.0043
GO biological process	GO:1901701	6	896	0.0044
GO biological process	GO:0001893	2	31	0.0045
GO biological process	GO:0001975	2	31	0.0045
GO biological process	GO:0008277	3	142	0.0045
GO biological process	GO:0019228	2	31	0.0045
GO biological process	GO:0042311	2	31	0.0045
GO biological process	GO:0062012	4	332	0.0045
GO biological process	GO:0045745	2	32	0.0047
GO biological process	GO:0051482	2	32	0.0047
GO biological process	GO:0002520	5	601	0.0048
GO biological process	GO:0007618	2	33	0.0049
GO biological process	GO:0010212	3	149	0.0049
GO biological process	GO:0032228	2	33	0.0049
GO biological process	GO:0034105	2	33	0.0049
GO biological process	GO:0043065	5	604	0.0049
GO biological process	GO:0051094	7	1286	0.0049
GO biological process	GO:0051590	2	33	0.0049
GO biological process	GO:0071312	2	33	0.0049
GO biological process	GO:1901568	3	148	0.0049
GO biological process	GO:0014074	3	151	0.0051
GO biological process	GO:0035239	5	615	0.0052
GO biological process	GO:0003006	5	622	0.0053
GO biological process	GO:0030501	2	35	0.0053
GO biological process	GO:0034405	2	35	0.0053
GO biological process	GO:0048738	3	154	0.0053
GO biological process	GO:0051223	5	622	0.0053
GO biological process	GO:0009266	3	155	0.0054
GO biological process	GO:0048519	14	4953	0.0054
GO biological process	GO:0051098	4	356	0.0054
GO biological process	GO:0051701	3	156	0.0054
GO biological process	GO:0019048	2	36	0.0055
GO biological process	GO:0032570	2	36	0.0055
GO biological process	GO:0086004	2	36	0.0055
GO biological process	GO:0006109	3	158	0.0056

GO biological process	GO:0097193	3	158	0.0056
GO biological process	GO:0086091	2	37	0.0057
GO biological process	GO:0021543	3	163	0.0059
GO biological process	GO:0045935	8	1770	0.0059
GO biological process	GO:0006928	7	1355	0.006
GO biological process	GO:0048167	3	164	0.006
GO biological process	GO:0007566	2	39	0.0061
GO biological process	GO:0001763	3	169	0.0064
GO biological process	GO:0046849	2	40	0.0064
GO biological process	GO:0051281	2	40	0.0064
GO biological process	GO:0051402	2	40	0.0064
GO biological process	GO:0048514	4	381	0.0065
GO biological process	GO:0048523	13	4454	0.0066
GO biological process	GO:0050796	3	172	0.0066
GO biological process	GO:0030522	3	173	0.0067
GO biological process	GO:0010628	8	1826	0.0068
GO biological process	GO:0042307	2	42	0.0068
GO biological process	GO:0048583	12	3882	0.0068
GO biological process	GO:0065009	11	3322	0.0068
GO biological process	GO:0048678	2	43	0.007
GO biological process	GO:0048732	4	395	0.007
GO biological process	GO:1903170	2	43	0.007
GO biological process	GO:0050679	3	178	0.0071
GO biological process	GO:0031328	8	1846	0.0072
GO biological process	GO:0050731	3	180	0.0072
GO biological process	GO:0048699	7	1422	0.0073
GO biological process	GO:0007268	4	402	0.0074
GO biological process	GO:0008542	2	45	0.0074
GO biological process	GO:0048608	4	405	0.0075
GO biological process	GO:1901654	3	183	0.0075
GO biological process	GO:0007626	3	184	0.0076
GO biological process	GO:0051209	2	46	0.0076
GO biological process	GO:2000026	8	1876	0.0077
GO biological process	GO:0055010	2	48	0.0081
GO biological process	GO:0097194	2	48	0.0081
GO biological process	GO:1903727	2	48	0.0081
GO biological process	GO:0001933	4	422	0.0084
GO biological process	GO:0035094	2	49	0.0084
GO biological process	GO:0050708	4	422	0.0084
GO biological process	GO:0032989	5	720	0.0085
GO biological process	GO:0048585	7	1483	0.0088
GO biological process	GO:0032101	5	732	0.009
GO biological process	GO:0050793	9	2416	0.0091
GO biological process	GO:0030099	3	203	0.0094
GO biological process	GO:0042981	7	1501	0.0094
GO biological process	GO:0051241	6	1098	0.0094
GO biological process	GO:0051345	5	742	0.0094
GO biological process	GO:0060688	2	53	0.0094
GO biological process	GO:0045944	6	1104	0.0095
GO biological process	GO:2000027	3	207	0.0098
GO biological process	GO:0007584	3	208	0.0099

GO biological process	GO:0030334	5	753	0.0099
GO biological process	GO:0032922	2	55	0.0099
GO biological process	GO:0030335	4	452	0.0101
GO biological process	GO:0006725	13	4754	0.0104
GO biological process	GO:0042130	2	57	0.0104
GO biological process	GO:0045600	2	59	0.0109
GO biological process	GO:0071242	2	60	0.0113
GO biological process	GO:0009968	6	1160	0.0116
GO biological process	GO:0071900	4	488	0.0128
GO biological process	GO:0045926	3	235	0.013
GO biological process	GO:0000904	4	498	0.0136
GO biological process	GO:0007169	4	499	0.0136
GO biological process	GO:0050795	2	68	0.0138
GO biological process	GO:0048646	5	831	0.014
GO biological process	GO:0040014	2	69	0.0141
GO biological process	GO:0032409	3	246	0.0144
GO biological process	GO:0008630	2	71	0.0147
GO biological process	GO:1901360	13	4963	0.0147
GO biological process	GO:0038127	2	72	0.015
GO biological process	GO:0045786	4	517	0.015
GO biological process	GO:0046889	2	73	0.0152
GO biological process	GO:0030097	4	526	0.0158
GO biological process	GO:0019233	2	75	0.0159
GO biological process	GO:0021536	2	75	0.0159
GO biological process	GO:0021766	2	75	0.0159
GO biological process	GO:0032943	2	76	0.0162
GO biological process	GO:0007416	2	77	0.0165
GO biological process	GO:0042475	2	78	0.0169
GO biological process	GO:0071260	2	78	0.0169
GO biological process	GO:0007166	8	2198	0.0171
GO biological process	GO:0055007	2	79	0.0172
GO biological process	GO:0007399	8	2206	0.0175
GO biological process	GO:0006869	3	272	0.0179
GO biological process	GO:0090068	3	273	0.0181
GO biological process	GO:0070542	2	82	0.0183
GO biological process	GO:1901570	2	82	0.0183
GO biological process	GO:0110110	2	83	0.0185
GO biological process	GO:0045597	5	908	0.0187
GO biological process	GO:0008585	2	84	0.0188
GO biological process	GO:0009892	9	2762	0.0188
GO biological process	GO:0048145	2	84	0.0188
GO biological process	GO:0048640	2	84	0.0188
GO biological process	GO:0007193	2	85	0.0191
GO biological process	GO:0071158	2	85	0.0191
GO biological process	GO:0098869	2	86	0.0194
GO biological process	GO:0016032	4	571	0.0197
GO biological process	GO:0010506	3	286	0.0198
GO biological process	GO:0042133	2	87	0.0198
GO biological process	GO:0032103	3	291	0.0206
GO biological process	GO:0006631	3	294	0.021
GO biological process	GO:0021782	2	91	0.0211

GO biological process	GO:0001655	3	299	0.0219
GO biological process	GO:0060548	5	953	0.0219
GO biological process	GO:0007631	2	94	0.0222
GO biological process	GO:2000379	2	94	0.0222
GO biological process	GO:0048638	3	302	0.0223
GO biological process	GO:0022603	5	961	0.0224
GO biological process	GO:0043112	2	95	0.0225
GO biological process	GO:0046677	3	305	0.0228
GO biological process	GO:0022607	8	2343	0.0232
GO biological process	GO:0051260	3	312	0.0241
GO biological process	GO:0006606	2	101	0.0251
GO biological process	GO:0032411	2	101	0.0251
GO biological process	GO:0002262	2	102	0.0254
GO biological process	GO:0032091	2	102	0.0254
GO biological process	GO:0007276	4	632	0.0263
GO biological process	GO:0032526	2	104	0.0263
GO biological process	GO:0042770	2	104	0.0263
GO biological process	GO:0033138	2	105	0.0265
GO biological process	GO:0032269	5	1014	0.0271
GO biological process	GO:0003014	2	107	0.0273
GO biological process	GO:0051781	2	107	0.0273
GO biological process	GO:0008203	2	109	0.0281
GO biological process	GO:0030855	4	649	0.0284
GO biological process	GO:0033365	4	649	0.0284
GO biological process	GO:0032970	3	338	0.0289
GO biological process	GO:0048589	3	340	0.0292
GO biological process	GO:0071902	3	340	0.0292
GO biological process	GO:0031324	8	2463	0.0297
GO biological process	GO:0031331	3	343	0.0297
GO biological process	GO:0040008	4	663	0.03
GO biological process	GO:0032147	3	347	0.0304
GO biological process	GO:0050864	2	118	0.0317
GO biological process	GO:1903532	3	364	0.0341
GO biological process	GO:0051222	3	365	0.0343
GO biological process	GO:0022407	3	366	0.0345
GO biological process	GO:0030326	2	126	0.0354
GO biological process	GO:0010605	8	2558	0.0358
GO biological process	GO:0006979	3	373	0.0359
GO biological process	GO:0062013	2	129	0.0365
GO biological process	GO:0035304	2	130	0.037
GO biological process	GO:1905330	2	130	0.037
GO biological process	GO:0044092	5	1119	0.0379
GO biological process	GO:0009749	2	133	0.0385
GO biological process	GO:0001819	3	390	0.0399
GO biological process	GO:0050777	2	136	0.0399
GO biological process	GO:0051254	6	1596	0.0406
GO biological process	GO:0040011	5	1144	0.0408
GO biological process	GO:0046683	2	138	0.0408
GO biological process	GO:0072594	3	396	0.0412
GO biological process	GO:1902107	2	139	0.0412
GO biological process	GO:0009888	6	1626	0.0437

GO biological process	GO:0045931	2	144	0.0438
GO biological process	GO:0006629	5	1192	0.0473
GO biological process	GO:2001257	2	152	0.0479
GO biological process	GO:0007093	2	153	0.0485
GO biological process	GO:0002683	3	425	0.0487
GO biological process	GO:0008152	18	9569	0.0487
GO biological process	GO:0001894	2	155	0.0493
KEGG pathway	hsa04726	9	112	2.65E-12
KEGG pathway	hsa04020	9	179	7.44E-11
KEGG pathway	hsa04261	8	139	3.92E-10
KEGG pathway	hsa04080	8	272	5.04E-08
KEGG pathway	hsa04270	6	119	2.80E-07
KEGG pathway	hsa04022	6	160	1.27E-06
KEGG pathway	hsa04970	5	86	1.99E-06
KEGG pathway	hsa05222	5	92	2.40E-06
KEGG pathway	hsa04024	6	195	2.64E-06
KEGG pathway	hsa05161	5	142	1.53E-05
KEGG pathway	hsa05223	4	66	2.37E-05
KEGG pathway	hsa05200	7	515	3.53E-05
KEGG pathway	hsa05210	4	85	5.26E-05
KEGG pathway	hsa04540	4	87	5.33E-05
KEGG pathway	hsa04933	4	98	7.84E-05
KEGG pathway	hsa04215	3	31	9.76E-05
KEGG pathway	hsa04919	4	115	0.00013
KEGG pathway	hsa04728	4	128	0.00018
KEGG pathway	hsa04010	5	293	0.00025
KEGG pathway	hsa05012	4	142	0.00025
KEGG pathway	hsa04723	4	148	0.00027
KEGG pathway	hsa04921	4	149	0.00027
KEGG pathway	hsa04932	4	149	0.00027
KEGG pathway	hsa05014	3	50	0.00027
KEGG pathway	hsa04923	3	53	0.00028
KEGG pathway	hsa05010	4	168	0.00035
KEGG pathway	hsa04370	3	59	0.00036
KEGG pathway	hsa05152	4	172	0.00036
KEGG pathway	hsa04924	3	63	0.0004
KEGG pathway	hsa05031	3	65	0.00042
KEGG pathway	hsa05167	4	183	0.00042
KEGG pathway	hsa04115	3	68	0.00045
KEGG pathway	hsa01524	3	70	0.00048
KEGG pathway	hsa05212	3	74	0.00054
KEGG pathway	hsa05410	3	81	0.00068
KEGG pathway	hsa04911	3	84	0.00074
KEGG pathway	hsa04727	3	88	0.00082
KEGG pathway	hsa04912	3	88	0.00082
KEGG pathway	hsa05414	3	88	0.00082
KEGG pathway	hsa04925	3	93	0.00089
KEGG pathway	hsa05146	3	94	0.00089
KEGG pathway	hsa05145	3	109	0.0013
KEGG pathway	hsa04725	3	111	0.0014
KEGG pathway	hsa04210	3	135	0.0023

KEGG pathway	hsa05206	3	149	0.0029
KEGG pathway	hsa05226	3	147	0.0029
KEGG pathway	hsa04960	2	37	0.0035
KEGG pathway	hsa05216	2	37	0.0035
KEGG pathway	hsa05225	3	163	0.0036
KEGG pathway	hsa05016	3	193	0.0056
KEGG pathway	hsa05030	2	49	0.0056
KEGG pathway	hsa05205	3	195	0.0056
KEGG pathway	hsa05134	2	54	0.0064
KEGG pathway	hsa05416	2	56	0.0067
KEGG pathway	hsa05213	2	58	0.007
KEGG pathway	hsa00590	2	61	0.0076
KEGG pathway	hsa04927	2	63	0.0079
KEGG pathway	hsa04720	2	64	0.008
KEGG pathway	hsa05214	2	68	0.0089
KEGG pathway	hsa05140	2	70	0.0092
KEGG pathway	hsa05412	2	72	0.0096
KEGG pathway	hsa04260	2	76	0.0104
KEGG pathway	hsa05220	2	76	0.0104
KEGG pathway	hsa01521	2	78	0.0106
KEGG pathway	hsa04742	2	81	0.0112
KEGG pathway	hsa05032	2	91	0.0138
KEGG pathway	hsa04657	2	92	0.0139
KEGG pathway	hsa04713	2	93	0.0139
KEGG pathway	hsa04750	2	92	0.0139
KEGG pathway	hsa04659	2	102	0.0159
KEGG pathway	hsa05165	3	317	0.0159
KEGG pathway	hsa04668	2	108	0.0175
KEGG pathway	hsa04724	2	112	0.0185
KEGG pathway	hsa04071	2	116	0.0193
KEGG pathway	hsa04151	3	348	0.0193
KEGG pathway	hsa04650	2	124	0.0215
KEGG pathway	hsa04926	2	130	0.0232
KEGG pathway	hsa04915	2	133	0.0239
KEGG pathway	hsa05034	2	142	0.0266
KEGG pathway	hsa05224	2	147	0.0281
KEGG pathway	hsa04934	2	153	0.0299
KEGG pathway	hsa05164	2	168	0.0351
KEGG pathway	hsa05202	2	169	0.0351
KEGG pathway	hsa05203	2	183	0.0401
KEGG pathway	hsa04015	2	203	0.0479
